# Exploratory Assessment of Geographical Variation in Metabolomic Profiles and In Vitro Bioactivities of Wild *Schisandra chinensis* Berries from the Changbai Mountains, China

**DOI:** 10.3390/foods15213941

**Published:** 2026-11-03

**Authors:** Peng Zhang, Yiyang Huang, Yan Wu, Xinman Liu, Yingying Zhang, Fangping Liu

**Affiliations:** 1 College of Veterinary Medicine, Northeast Agricultural University, Harbin 150030, China; 18741363690@163.com (P.Z.);; 2 College of Life Engineering, Shenyang Institute of Technology, Fushun 113122, China; 3 College of Animal Science and Veterinary Medicine, Shenyang Agricultural University, Shenyang 110866, China; 4 Heilongjiang Key Laboratory for Animal Disease Control and Pharmaceutical Development, Harbin 150030, China

**Keywords:** *Schisandra chinensis* (Turcz.) Baill., geographical origin, biological activity, enzyme inhibition, species distribution modeling

## Abstract

*Schisandra chinensis* (Turcz.) Baill. is widely recognized as a functional berry, yet systematic evaluations of its metabolomic variations and bioactivities across different growing regions remain limited. Berries collected from eight regions in the Changbai Mountains were analyzed using Q-Orbitrap LC-MS-based metabolomics. We identified 18 differential metabolites, with SC1 (Kuandian) exhibiting the highest total lignan content (13.713 mg/g DW). Soils were acidic with variable nutrients; MaxEnt modeling predicted Liaoning as the most suitable habitat. In vitro bioactivities showed considerable variation by origin: SC1 showed the most favorable antioxidant trend; SC7 (Yanbian) demonstrated relatively strong anti-inflammatory and acetylcholinesterase inhibition; SC6 (Baishan) exhibited the most favorable α-amylase inhibition; SC8 (Ning’an) displayed relatively strong α-glucosidase inhibition; and SC3/SC4 (Xiuyan/Xinbin) had the most favorable hepatoprotective effects. Geographic origin is associated with variations in metabolite accumulation and bioactivity, with soil properties and climatic factors showing correlations with these parameters, offering a preliminary basis for quality evaluation and origin prioritization.

## 1. Introduction

Modern diets high in sugar and fat have contributed to a rise in various health issues, driving interest in effective prevention and management strategies [1,2]. Common problems include dysregulated blood glucose and lipid concentrations, oxidative stress, and chronic inflammation, which involve multiple biological pathways [3,4]. Natural bioactive compounds from plant-based foods—flavonoids, polysaccharides, and saponins—have attracted attention as functional food ingredients due to their multi-target activities [5,6,7].

*Schisandra chinensis* (Turcz.) Baill., a perennial vine of the Schisandraceae family, is valued as both a medicinal and edible berry and has been developed into dietary supplements [8,9]. Its fruits contain lignans, polysaccharides, volatile organic compounds (VOCs), and organic acids [10], among which lignans (e.g., schisandrin A, schisandrol A, gomisin) are considered the most important secondary metabolites and exhibit antioxidant, anti-inflammatory, and hepatoprotective activities [9]. This species has a long history of traditional use as both food and medicine in northeastern Asia. Fresh berries are tart and are often mixed with sweeter fruit to balance the flavor, while dried berries are steeped in hot water and consumed as a tea. In Korean tradition, the berries are the primary ingredient in “omija hwachae”, a chilled punch served during hot summer days, and were formulated into “Omigalsu” [11], a beverage documented in the UNESCO Memory of the World-registered Dongeuibogam for treating “sogal”, a condition resembling diabetes mellitus [12]. In the Russian Far East, Nanai hunters consumed dried *Schisandra* berries as a tonic to combat hunger, thirst, and exhaustion during multi-day sable hunts [13]. Among the Oroqen people of northeastern China, a decoction of the berries is prepared as a welcoming tea valued for its refreshing effect and its use in treating neurasthenia. These diverse traditional uses—as a fresh fruit, a processed beverage, a stimulant tonic, and a medicinal tea—provide the ethnobotanical foundation for modern investigations into the berry’s functional properties. However, lignan accumulation and bioactivity are strongly influenced by environmental factors such as soil and climate [14,15]. This variability poses a challenge to developing *S. chinensis* as a standardized, high-value functional food ingredient.

Overharvesting has led to a progressive decline in wild *S. chinensis* resources [16]. This sustainability issue is intertwined with industrial constraints, including price volatility, supply–demand imbalances, and low production standardization. Therefore, geographical authentication and quality standardization technologies are urgently needed for the sustainable development of the *Schisandra chinensis* industry. Mass spectrometry-based metabolomic fingerprinting has been successfully used to discriminate *S. chinensis* from different origins [17], providing technical support for traceability systems and quality assurance of functional food ingredients. By elucidating chemical profiles from different origins, spatially and temporally specific harvesting strategies can be developed to prevent depletion of high-quality wild resources [18]. Moreover, identifying key environmental factors that affect metabolite accumulation enables precise “origin-quality” matching, facilitating the selection of optimal growing regions or the refinement of cultivation conditions [19]. Collectively, these geographical authentication technologies can promote industrial sustainability and help transform S. chinensis from a traditional Chinese medicinal herb into a standardized functional food ingredient.

The Changbai Mountain range is a major ecological region in northeastern China and a primary habitat for wild *S. chinensis* [20]. In recent years, increased market demand and habitat alteration have put pressure on wild resources [21]. Although many studies have focused on specific components and activities of *Schisandra chinensis* [19], comprehensive research linking overall metabolite differences to bioactivity across different areas of the Changbai Mountains remains lacking. In particular, to our knowledge, no study has integrated metabolomic profiling, targeted lignan quantification, habitat suitability modeling, rhizosphere soil analysis, and multiple in vitro bioassays within a single sampling framework for wild *S. chinensis* in this region.

To identify favorable growing regions for high-quality *S. chinensis*, we performed targeted and untargeted metabolomic profiling of samples from different areas within the Changbai Mountain range. We also used remote sensing and trace element analysis of root-zone soil to investigate environmental influences on the metabolome at both macro- and micro-levels. Furthermore, we systematically evaluated in vitro bioactivities, including antioxidant, anti-inflammatory, and hypoglycemic effects. This study aims to test the hypothesis that geographical origin-associated variations in soil properties and climate are associated with differential accumulation of lignans, which in turn are associated with the bioactivity profiles of *S. chinensis*. The findings will support the consistent quality and efficacy of this functional food ingredient, with direct implications for phytochemistry and the health industry.

## 2. Materials and Methods

### 2.1. Reagents

Schisandrol A, schisandrol B, Schisandrin A, Schisandrin B, Angeloyl gomisin H, Gomisin J, Schisanhenol, and Schisantherin A (HPLC purity > 98%) were obtained from Hubei Cuiyuan Biotechnology Co., Ltd. (Wuhan, Hubei, China). The reagents 2,2-diphenyl-1-picrylhydrazyl (DPPH) and 2,2′-azino-bis(3-ethylbenzothiazoline-6-sulfonic acid) (ABTS) were sourced from Aladdin Co. (Shanghai, China). Ethanol, petroleum ether, ethyl acetate, n-butanol, methanol (gradient grade, ≥99.9%), acetonitrile (gradient grade, ≥99.9%), and formic acid (gradient grade, ≥95%) were procured from Beijing Reagent Co., Ltd. (Beijing, China). α-Glucosidase (S10050), acetylcholinesterase (S33095), acarbose (≥98%, B20003), LPS (S55571), and APAP (≥99%, S31044) were obtained from Shanghai Yuanye Bio-Technology Co., Ltd. (Shanghai, China). α-Amylase (G8290) and soluble starch (G8300) were sourced from Beijing Solarbio Science & Technology Co., Ltd. (Beijing, China). DTNB (≥99%, A146608) and galantamine (≥99%, A697166) were acquired from AmBeed (Beijing, China). The Cell Counting Kit-8 (G4103) was purchased from Wuhan Servicebio Technology Co., Ltd. (Wuhan, China). The Aspartate aminotransferase Assay Kit (C010-2-1) and Alanine aminotransferase Assay Kit (C009-2-1) were obtained from Nanjing Jiancheng Bioengineering Institute (Nanjing, China). The mouse RAW 264.7 macrophage cell line and AML12 hepatocyte cell line were sourced from the American Type Culture Collection (ATCC, Manassas, VA, USA). DMEM-F12 medium was acquired from Gibco (Grand Island, NY, USA).

### 2.2. Plant Materials and Sample Preparation

Fresh fruit samples were collected from eight sampling sites in three provinces during September–October 2024 (Table 1). Voucher specimens (accession numbers SC1–SC8) were authenticated by Professor Junfan Fu, College of Life Engineering, Shenyang Institute of Technology. Due to the sparse distribution of wild *Schisandra chinensis* resources and restrictions on sampling permits in protected areas, a single representative composite sample was collected from each of the eight sampling sites. At each site, fruits were harvested from 20 spatially separated individual plants (distance >10 m between plants to minimize clonal relatedness), and approximately 300 g of fresh fruits was pooled to form one composite sample per location. The composite samples were immediately rinsed with distilled water, frozen in liquid nitrogen at the collection sites, and stored at −80 °C until extraction. All subsequent chemical and bioactivity analyses rely solely on technical triplicates rather than independent biological replicates, which cannot quantify the biological variation among individual plants within a single location. Statistical comparisons across sampling sites are therefore interpreted as exploratory descriptive trends rather than definitive biological differences. We utilized the Global Positioning System (GPS) to record essential geographical information, including longitude, latitude, and altitude. Rhizosphere soil (0–20 cm depth) was sampled from fruit-bearing *Schisandra chinensis* plants. At each site, soil totaling around 500 g was gathered from three random positions within a 1 m radius of each of three target plants and homogenized into a single composite sample. After removing stones and obvious plant debris, soils were air-dried at 25 ± 2 °C at room temperature, pulverized and sieved with a 0.149 mm mesh for measurement. This pretreatment method refers to the standard specifications for rhizosphere soil detection in medicinal plant research [22].

### 2.3. Preparation of Schisandra Chinensis Extract

According to Zhang’s method, we weighed 3 g of *Schisandra chinensis* powder and placed it into a 250 mL conical flask [23]. Subsequently, 100 mL of 80% ethanol was added, and the mixture was allowed to soak for 24 h. The extraction was then conducted using ultrasonic power at 550 W and a temperature of 40 °C for 30 min, followed by centrifugation at 10,000 rpm for 5 min to collect the precipitate. An additional 100 mL of 80% ethanol was added, and the aforementioned steps were repeated for a total of three extractions. The resulting filtrates were combined, rotary-evaporated at 40 °C, and subsequently vacuum freeze-dried to yield *Schisandra chinensis* extract (SCE).

### 2.4. Analysis of the Differences in Secondary Metabolites of Schisandra chinensis in Different Provinces

#### 2.4.1. Detection Conditions for Q-Orbitrap High-Resolution Liquid Chromatography–Mass Spectrometry

According to Xie et al., 100 mg of *Schisandra chinensis* was weighed and mixed with 1 mL of 80% methanol using vortexing [24]. Subsequently, 2–3 zirconium dioxide grinding beads were added, followed by 5 min of grinding extraction and 10 min of vortex mixing. The mixture was then centrifuged at 1513× *g* for 10 min at 4 °C. The supernatant was filtered through a 0.22 μm membrane, and the resulting filtrate was collected for instrumental analysis. LC-MS analysis was performed on a Q-Exactive Orbitrap mass spectrometer coupled with an Ultimate 3000 UHPLC system (Thermo Fisher Scientific, Waltham, MA, USA). Chromatographic separation was achieved on an HSS T3 column (100 mm × 2.1 mm, 1.8 μm, Waters, Milford, MA, USA) at 35 °C with a flow rate of 0.3 mL/min. The mobile phases were (A) water with 0.1% formic acid and (B) methanol with 0.1% formic acid. The gradient program was as follows: 0–1 min, 95% A; 1–8 min, 70% A; 8–15 min, 40% A; 15–22 min, 2% A; 22–28 min, 2% A; 28–28.5 min, 95% A; 28.5–30 min, 95% A. The injection volume was 5 μL. Mass spectrometry was performed in positive and negative ion modes with the following parameters: spray voltage 3.8 kV (positive)/3.2 kV (negative); capillary temperature 300 °C; sheath gas flow 40 Arb; auxiliary gas flow 15 Arb; resolution 70,000 for full scan and 17,500 for dd-MS^2^. The mass spectrometer was externally calibrated every three days using the Thermo Scientific Pierce™ Calibration Solution (Thermo Fisher Scientific, Waltham, MA, USA), and mass accuracy was maintained within 2 ppm throughout the analytical sequence.

#### 2.4.2. Data Processing and Analysis

Raw data were processed using Compound Discoverer 3.3 (Thermo Fisher). A pooled quality control (QC) sample was prepared by mixing 100 μL of the reconstituted extract from each individual sample in equal volumes. One QC injection was performed after every 10 sample injections to monitor instrument stability. Features with a coefficient of variation (CV) > 30% in QC samples were excluded from further analysis. Peak intensities were normalized by total ion current (TIC) within each injection. Before multivariate analysis, data were Pareto-scaled (mean-centered and divided by the square root of the standard deviation). Peak deconvolution was performed using the adaptive wavelet transform algorithm (peak width range 5–30 s), with a minimum peak intensity corresponding to S/N > 100; peak alignment used the Obiwarp algorithm (bins = 100, gapInit = 0.5).

Compound annotation was performed by matching retention time, precursor ion mass, and MS/MS spectra against mzCloud, mzVault and ChemSpider. Within the Compound Discoverer 3.3 software platform, preprocessing steps included peak identification, retention time correction, and automatic integration. The peak area threshold (1 × 10^8^) corresponds to S/N > 100, ensuring reliable detection. VIP > 1 is standard for selecting discriminatory variables. The *p* < 0.05 threshold was applied after FDR correction (Benjamini–Hochberg, q < 0.05). Data analysis was conducted using SIMCA 14.1 software, which employed principal component analysis (PCA) and orthogonal partial least squares-discriminant analysis (OPLS-DA), along with S-plot analysis to examine metabolic differences and identify potential marker compounds across three provinces (A, B, and C) [25]. To evaluate model overfitting, a permutation test with 200 random permutations was performed for the OPLS-DA model.

### 2.5. Detection of Lignan Content in Schisandra chinensis Extracts from Different Regions

The LC-MS/MS method was utilized to analyze nine common lignans present in SCE from various regions, including Schisandrol A, schisandrol B, Schisandrin A, Schisandrin B, Angeloyl gomisin H, Gomisin J, Schisanhenol, and schisantherin A [26]. A 1 g sample was weighed and combined with 30 mL of methanol in a stoppered conical flask. The mixture was subjected to ultrasonic extraction at 60 kHz and 250 W for three cycles, with each extraction lasting 20 min. The analysis was conducted using the Thermo Vanquish ultra-high-performance liquid chromatography system in conjunction with the Thermo Scientific Quantis LC-MS/MS system, employing a Waters ACQUITY UPLC^®^ C18 column (2.1 × 50 mm, 1.7 μm). Ionization was achieved using an electrospray ionization (ESI) source in positive ion mode, with monitoring performed in multiple reaction monitoring (MRM) mode. The electrospray voltage was set at 2.5 kV, the nebulizer temperature at 350 °C, and the ion transfer tube temperature at 325 °C, with sheath gas and auxiliary gas flows maintained at 50 Arb and 10 Arb, respectively. The mobile phase consisted of A (water containing 0.1% formic acid) and B (acetonitrile containing 0.1% formic acid) in a ratio of 45:55, with a flow rate of 0.3 mL/min and an injection volume of 1 μL. The mass spectrometry parameters for each component are detailed in Supplementary Table S1. Each sample was analyzed in triplicate, and the main peak identification, standard solution preparation, and methodological validation were conducted following the protocol established by Wang et al. [27].

### 2.6. Study on Growth Adaptability Zoning of Wild Schisandra chinensis in Changbai Mountain Area

#### 2.6.1. Research Area and Data Sources

**Figure 1:**
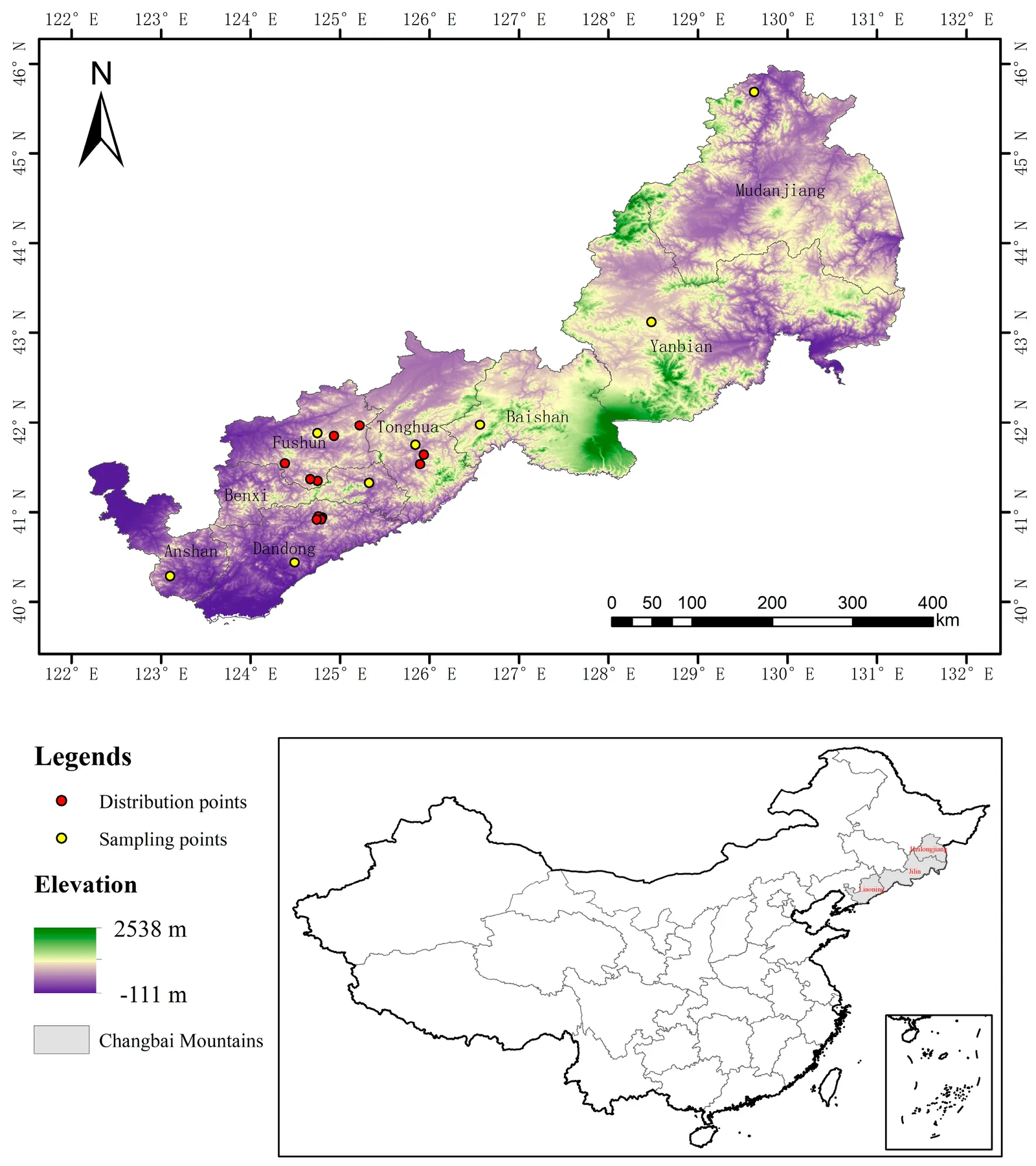
Geographical distribution map of wild *Schisandra chinensis* in the Changbai Mountain Region. Base map and administrative boundaries from the National Geographic Information Resource Directory Service System (1:1,000,000 public version, 2021). Elevation and vegetation background from the Resource and Environment Science and Data Center.

Field sampling and online data retrieval were conducted using platforms such as the Global Biodiversity Information Facility (GBIF, https://www.gbif.org/, accessed on 10 September 2025), the Chinese Virtual Herbarium (CVH, http://www.cvh.ac.cn/, accessed on 10 September 2025), and the National Specimen Information Infrastructure (NSII, https://www.nsii.org.cn/2017/, accessed on 10 September 2025). During this process, duplicate locations, overlapping coordinates, and uncertain specimen records were eliminated. Ultimately, a total of 20 sampling points were obtained for model construction (Figure 1, Table S2) [28]. It should be noted that despite comprehensive searches in GBIF, CVH, and NSII, no additional wild *S. chinensis* occurrence records with sufficient spatial accuracy could be obtained for the study area, largely due to the species’ sparse distribution and restricted access to certain protected zones. Therefore, the following MaxEnt modeling should be considered an exploratory analysis based on the best available data.

#### 2.6.2. Environmental Data

Climate variables were sourced from the WorldClim database (http://www.worldclim.org/), covering the period from 1970 to 2000, with a spatial resolution of 30 arc-seconds (approximately 1 km). A total of 19 bioclimatic variables, labeled bio-1 to bio-19, were included in the analysis. Soil variables were obtained from the World Soil Database (http://webarchive.iiasa.ac.at/Research/LUC/External-World-soil-database/HTML/, accessed on 10 September 2025). Based on the growth environment characteristics of *Schisandra chinensis*, 13 soil variables were selected, including base saturation (t-bs), carbonate or lime content (t-caco3), sulfate content (t-caso4), cation exchange capacity of the clayey layer (t-cec-clay), cation exchange capacity of the soil (t-cec-soil), clay content (t-clay), exchangeable sodium percentage (t-esp), gravel volume percentage (t-gravel), organic carbon content (t-oc), pH (t-ph-h2o), soil bulk density (t-ref-bulk), exchangeable bases (t-teb), and USDA soil texture classification (t-usda-tex). Additionally, vegetation type data were obtained from the Resource and Environment Science and Data Center. Elevation (alt) and slope aspect (aspect) derived from elevation data processing were also included.

#### 2.6.3. Map Data

The map data was sourced from the 1:1 million scale China map and the administrative division map, which were downloaded from the National Geographic Information Resource Directory Service System (https://www.webmap.cn/commres.do?method=dataDownload, accessed on 10 September 2025).

#### 2.6.4. Environmental Factor Screening

In the MaxEnt model, the significance of 58 environmental variables was initially assessed using the Jackknife test, leading to the exclusion of 41 variables that exhibited a contribution rate of 0. Subsequently, the Extract Values to Points tool in ArcGIS 10.4 was employed to extract data for 17 variables (including t-bs, t-teb, t-cec-soil, bio-8, bio-2, among others) at 20 distribution points. A Spearman correlation analysis was conducted on these variables using SPSS 26.0, resulting in the removal of one of the two variables with a correlation coefficient |r| ≥ 0.80, which culminated in the exclusion of 4 variables. Ultimately, 13 environmental variables were retained for further analysis.

#### 2.6.5. Prediction by the MaxEnt Model

Using MaxEnt software (version 3.4.1), we imported 20 distribution records along with 13 selected environmental factors. The test set comprised 25% of the distribution points, while the training set constituted 75% of the distribution points. In the environmental parameter settings, the Jackknife method was activated to assess the significance of each environmental factor. All other parameters were set to their default values: the regularization multiplier was 1.0; feature classes were set to “auto features”, which, given the limited number of occurrence points (*n* = 20), led the software to automatically select linear, quadratic, and product features while avoiding hinge and threshold features to minimize overfitting [29]. Background points were randomly drawn from the study area using the default number of 10,000 points. To assess model stability and predictive performance, 10-fold cross-validation (10 replicates) was performed, and the area under the receiver operating characteristic curve (AUC) was used as the primary threshold-independent evaluation metric [30]. Although small sample sizes can inflate AUC values [31], the cross-validated AUC still provides a relative measure of model discriminability.

#### 2.6.6. Model Accuracy Verification

The Receiver Operating Characteristic (ROC) curve is a comprehensive metric that reflects the continuous variables of model sensitivity and specificity, with the area under the curve (AUC) serving as an indicator of model accuracy. The AUC value ranges from 0 to 1, where values closer to 1 indicate better accuracy. An AUC between 0.6 and 0.8 suggests moderate predictive performance, while values between 0.8 and 0.9 indicate good predictive capability. An AUC exceeding 0.9 signifies high accuracy, thereby enabling a reliable representation of species’ potential distribution.

#### 2.6.7. Suitability Distribution Zoning

In ArcGIS 10.4, the results of the MaxEnt operation are loaded, and the generated raster data files are reclassified. Based on the habitat suitability index (*p* value), which ranges from low to high, the potential distribution areas of *Schisandra chinensis* are categorized into four grades, with *p* values of 0.6 and above indicating highly suitable habitats. The spatial analysis module is then utilized to calculate the area corresponding to each suitability grade, leading to the determination of *Schisandra chinensis*’s potential distribution results.

### 2.7. Detection of Soil Physical and Chemical Properties of Schisandra chinensis Roots in Different Regions

Soil pH was measured using a pH meter (soil:water = 1:2.5, *w*/*v*). Soil organic matter (SOM) was determined by the potassium dichromate external heating method (LY/T 1237-1999; detection limit: 0.2 g/kg). Total nitrogen (TN) was measured by the Kjeldahl digestion method (HJ 717-2014; detection limit: 48 mg/kg). Total phosphorus (TP) was analyzed by the alkali fusion–molybdenum antimony colorimetric method (LY/T 1232-2015; detection limit: 0.01 mg/kg). Available phosphorus (AP) was extracted with HCl-H_2_SO_4_ (soil:extractant = 1:5, *w*/*v*) at 180 rpm for 30 min at 25 °C (LY/T 1233-1999; detection limit: 0.5 mg/kg). Available potassium (AK) was extracted with 1 mol/L ammonium acetate (pH 7.0) and measured by flame photometry (NY/T 889-2004; detection limit: 0.5 mg/kg) [32].

For quality assurance, reagent blanks were run with every batch, 10% of samples were analyzed in duplicate (RSD consistently < 5%), and certified reference material GBW07461 was analyzed every 10 samples with recovery within 95–105%.

### 2.8. Evaluation of In Vitro Biological Activity of Schisandra chinensis Extracts in Different Regions

#### 2.8.1. Antioxidant Capacity Detection

DPPH and ABTS are two of the most commonly used free radicals in antioxidant assays. To assess the DPPH and ABTS radical scavenging capacities of SCE from various regions, we followed the methodology outlined by Marinaccio et al. [33]. SCE was dissolved in 80% ethanol to the tested concentrations. SCE samples were prepared at concentrations of 5, 10, 15, 20, 25, and 30 mg/mL. Subsequently, 3.8 mL of DPPH solution was combined with 200 μL of the sample, and the mixture was allowed to react in the dark for 30 min. The absorbance of the solution was then measured at a wavelength of 517 nm. For the ABTS assay, a 7 mmol/L solution was prepared using PBS buffer, and a 2.45 mmol/L solution was created with potassium persulfate. Following complete dissolution, the ABTS solution was combined with the potassium persulfate solution in equal volumes, and the resulting mixture was incubated in the dark at 4 °C for 16 h. SCE solutions at concentrations of 25, 50, 75, 100, and 125 μg/mL were then added, with VC serving as the control. Each sample was analyzed in triplicate. The absorbance was measured at 734 nm after a 7-min reaction period.

#### 2.8.2. In Vitro Anti-Inflammatory Activity Assay

Following the methodology outlined by Liu et al., an inflammatory model was established using RAW 264.7 macrophages [34]. Various extracts of *Schisandra chinensis*, sourced from different regions, were added to the culture plate at a concentration of 200 µg/mL and incubated for 1 h. The concentration of 200 µg/mL was selected based on preliminary cytotoxicity tests showing no significant reduction in RAW264.7 cell viability at concentrations ≤ 200 µg/mL. After this incubation, lipopolysaccharide (1 µg/mL) was introduced to the culture plate and allowed to incubate for an additional 24 h to facilitate the collection of the supernatant. Finally, 50 µL of the supernatant, 50 µL of Griess reagent I, and 50 µL of Griess reagent II were combined in the culture plate, and the absorbance was measured at 540 nm using a microplate reader. Each sample was analyzed in triplicate.

#### 2.8.3. Evaluation of In Vitro Hepatoprotective Effects

The method described by Zhou et al. employed the CCK8 assay to evaluate the effects of SCE from eight different regions on the viability of AML12 cells [35]. The concentration of 100 µg/mL was chosen based on preliminary CCK-8 assays demonstrating no cytotoxicity to AML12 cells at this level. Cell suspensions were introduced into 96-well plates, followed by the addition of 100 μg/mL SCE from each of the eight regions. After a 2-h treatment period, 2.5 mmol/L APAP was added, and the cells were incubated for an additional 48 h. Subsequently, the CCK8 reagent was utilized to assess the impact of SCE on the viability of both AML12 cells and APAP-damaged AML12 cells. Concurrently, the levels of ALT and AST in the cell supernatant were measured. ALT and AST activities in the culture supernatant were determined using commercial assay kits (Nanjing Jiancheng) according to the manufacturer’s instructions; absorbance was measured at 510 nm and 505 nm, respectively, and activities were expressed as U/L. Each sample was analyzed in triplicate.

### 2.9. Enzyme Activity Inhibition Experiment

The inhibitory effects of SCE (5, 6, 7, 8, 9 mg/mL) from various regions on α-glucosidase activity were assessed using the Marinaccio method, with acarbose serving as the positive control [33]. Absorbance values for each group were measured at a wavelength of 405 nm using a microplate reader. In a similar manner, the inhibitory effects of SCE (5, 6, 7, 8, 9 mg/mL) from different regions on α-amylase activity were evaluated, again using acarbose as the positive control, and absorbance values were recorded at 540 nm. Additionally, we investigated the inhibitory effects of SCE (0.09375, 0.1875, 0.375, 0.75, 1.5, 3 mg/mL) from various regions on AchE activity, employing galantamine as the positive control. Each sample was analyzed in triplicate. The absorbance values for each group were measured at 412 nm using a microplate reader.

### 2.10. Statistical Analysis

Statistical analyses were performed using GraphPad Prism 9 (GraphPad Software, San Diego, CA, USA) and R version 4.2.0 [36]. All data are presented as mean ± standard deviation (SD) of at least three independent technical replicates. Due to the exploratory sampling design (one composite sample per location), all statistical results should be interpreted with caution and considered hypothesis-generating rather than confirmatory. For lignan contents across the eight sampling sites (Table 3), only descriptive statistics (mean ± SD) are reported. Comparisons between treatment groups and their respective controls (e.g., LPS-treated vs. blank control; APAP-treated vs. control) were analyzed using one-way ANOVA followed by Dunnett‘s test, with significance set at *p* < 0.05. Comparisons among SCE samples from different regions are presented descriptively without significance markers, as these samples derive from the composite plant sampling design (*n* = 1 per location). It should be noted that soil samples were collected as three independent biological replicates (*n* = 3) per site, whereas plant samples were represented by one composite sample per site; therefore, the statistical treatments differ accordingly. Due to the significant spatial variation and non-normal distribution of soil elements (Shapiro–Wilk test, *p* < 0.05), the Kruskal–Wallis H test was used to compare the overall differences among the 8 treatment groups. When the overall test was significant, the Dunn test was used for post hoc pairwise comparisons, and the Bonferroni method was employed to correct the significance level of multiple comparisons (corrected α = 0.05) (Table 5). IC_50_ values for DPPH, ABTS, α-amylase, α-glucosidase, and acetylcholinesterase inhibition were calculated using GraphPad Prism 9 with non-linear regression (four-parameter logistic model). The 95% confidence intervals (CIs) for each IC_50_ are reported in Table 6. Correlation analyses between environmental/soil factors, lignan contents, and bioactivities (Figure 5A,B) were performed using Spearman’s rank correlation coefficient, with *p*-values adjusted for multiple comparisons using the Benjamini–Hochberg false discovery rate (FDR) method (q < 0.05 considered suggestive rather than definitive). Mantel tests (Figure 5C) were performed for exploratory purposes to assess associations between distance matrices derived from environmental variables, lignan contents, and bioactivities. Euclidean distances were calculated after data transformation (log-transformation for skewed variables and Z-score standardization). Significance was evaluated using 9999 permutations. Given the limited sample size, the Mantel test results should be interpreted as indicating potential trends rather than confirmed relationships.

## 3. Results and Discussion

### 3.1. Comparison of Chemical Components and Biomarkers of Schisandra chinensis in Different Provinces

The chemical components of SCE were acquired with LC-MS and aligned to different databases, including mzCloud, mzVault and ChemSpider, for characterization. To filter reliable features, peaks with a coefficient of variation (CV) > 30% in quality control (QC) samples were excluded. The remaining features were then subjected to orthogonal partial least squares-discriminant analysis (OPLS-DA). Variable importance in the projection (VIP) scores were extracted from the OPLS-DA model, and compounds with VIP > 1.0 were retained as important contributors to group discrimination. This threshold is widely recommended in metabolomics studies to select discriminative features while reducing false positives. Subsequently, *p*-values were adjusted for multiple comparisons using the Benjamini–Hochberg false discovery rate (FDR) correction, and only metabolites with an adjusted *p*-value < 0.05 were considered statistically significant. Additionally, to ensure analytical reliability, only features with a mean peak area exceeding 1 × 10^8^ (corresponding to a signal-to-noise ratio > 100) were retained. Applying these sequential filtering criteria led to the identification of 18 differential metabolites, including lignans, flavonoids, and polysaccharides (Table 2).

**Figure 2:**
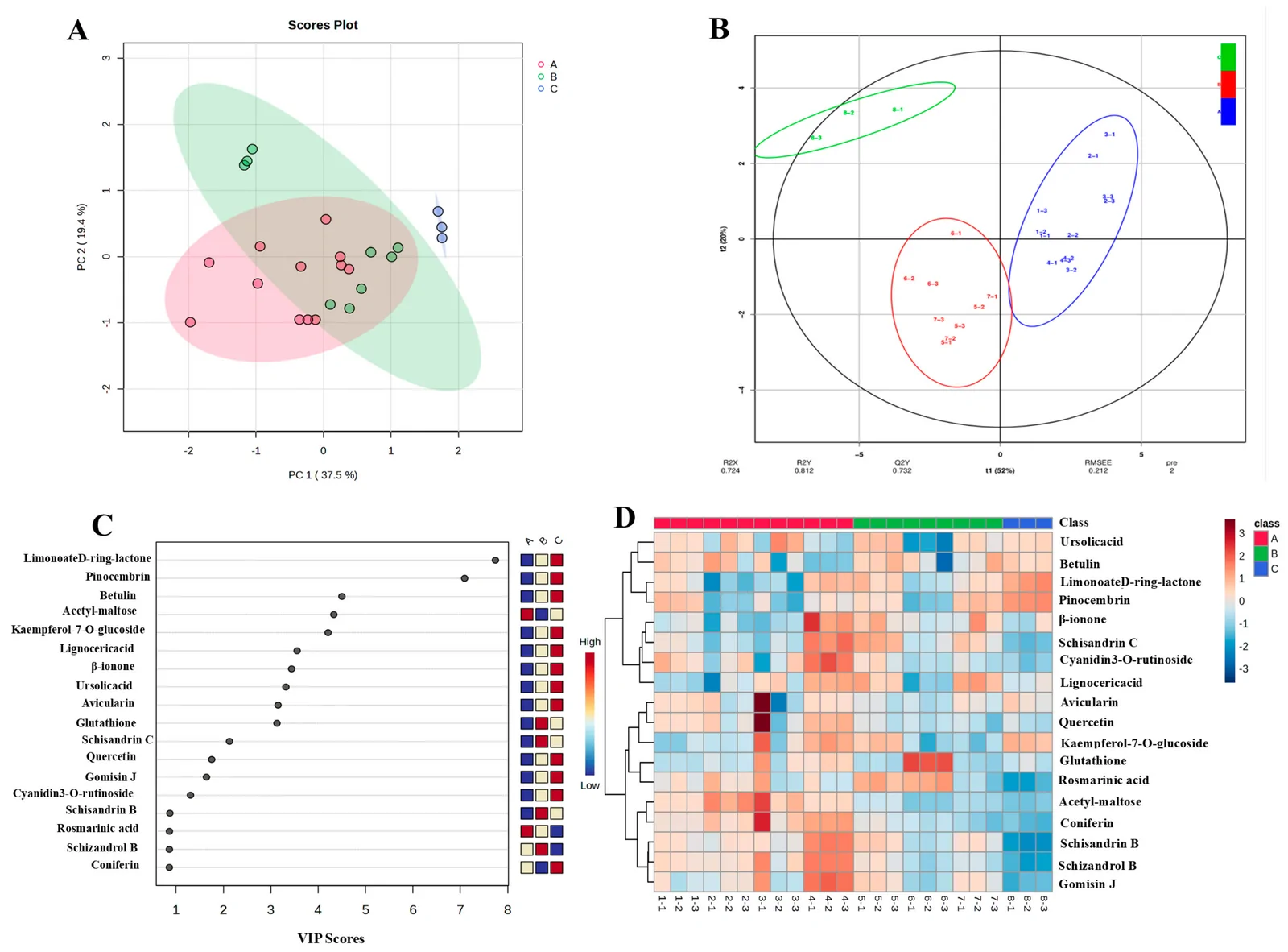
Differential analysis of secondary metabolites in *S. chinensis* from different regions. (**A**) PCA score plot of *S. chinensis* from three provinces (PC1 = 37.5%, PC2 = 19.4%, cumulative 56.9%), (**B**) OPLS-DA of *S. chinensis* from three provinces, (**C**) S-plot of secondary metabolites in *S. chinensis* from three provinces, (**D**) heatmap showing the relative abundance of 18 identified compounds in *S. chinensis* from different regions. Heilongjiang Province was represented by only one sampling site.

To comprehensively investigate the chemical differences among *Schisandra chinensis* samples from three provinces (A, B, and C), we employed Principal Component Analysis (PCA) and Partial Least Squares Discriminant Analysis (PLS-DA) to identify compositional variations across these regions [37,38]. PCA revealed observed inter-provincial differences in the present sample set; samples from regions A and B exhibited partial overlap while remaining clearly separated from region C, consistent with their geographical distribution (Figure 2A). By incorporating group information, the supervised OPLS-DA significantly enhanced inter-group separation [39], with a high goodness-of-fit (R^2^Y = 81.2%) and predictive capability (Q^2^Y = 73.2%), indicating that the differences between groups outweighed the variations within groups (Figure 2B). A permutation test (200 random permutations, Figure S1) gave a Q^2^ intercept of −0.196, confirming no overfitting. Variables with Variable Importance in Projection (VIP) scores greater than 1.0 were identified as potential discriminant metabolites (Figure 2C). Notably, Limonoate D-ring-lactone, Pinocembrin, Betulin, Kaempferol-7-O-glucoside, Lignoceric acid, β-ionone, and Cyanidin 3-O-rutinoside were found to be more abundant in region C (Heilongjiang Province). In contrast, Schisandrin C, Schizandrol B, Schisandrin B, and Glutathione were more prevalent in region B (Jilin Province). Acetyl-maltose and Rosmarinic acid exhibited relatively higher abundance in region A (Liaoning Province). To further elucidate the metabolite variations among cities, we conducted a HeatMap analysis (Figure 2D). The results indicated that Fushun City (SC4) possesses a richer array of secondary metabolites compared to other cities, including Schisandrin B, Schizandrol B, Schisandrin C, Cyanidin 3-O-rutinoside, β-ionone, and Gomisin J. Additionally, Glutathione was more abundant in Baishan City (SC6), while Acetyl-maltose was relatively more abundant in Benxi City (SC2) and Anshan City (SC3). Furthermore, Limonoate D-ring-lactone, Pinocembrin, and Kaempferol-7-O-glucoside showed relatively higher levels in Mudanjiang City (SC8). Given the limited sample sites (*n* = 8 composite samples), the observed clustering patterns should be interpreted as preliminary and hypothesis generating rather than definitive.

### 3.2. Determination of SCE Lignan Content by LC-MS/MS

The chromatogram of the mixed reference solution (Figure S2) demonstrated distinct separation of retention times for the nine lignans. A two-fold dilution method was employed to prepare solutions with varying mass concentrations from one milliliter of the reference stock solution, which were subsequently injected into the liquid chromatography system, and their chromatographic peak areas were recorded. By plotting the peak area (Y) against the reference mass concentration (X, ng/mL), linear regression analysis was conducted to derive the regression equations, linear ranges, and correlation coefficients for each component (Table S3). The precision of the analytical method was assessed through intra-day and inter-day variations, with results indicating good repeatability and high accuracy of the analytical conditions (Table S4). The contents of nine lignans in *Schisandra chinensis* extracts from eight different regions (SC1–SC8) were determined by LC-MS/MS (Table 3). Notable regional variations were observed for both individual and total lignan contents. Total lignan content ranged from 7.71 mg/g DW to 13.71 mg/g DW, with SC1 showing the highest value, followed by SC5 (13.12 mg/g DW). SC1 also showed the highest observed levels of schisandrol A (8.06 mg/g DW), schisandrol B (0.317 mg/g DW), and angeloylgomisin H (1.585 mg/g DW). For schisandrin B and gomisin D, SC5 had the highest concentrations (2.382 and 0.369 mg/g DW, respectively). Notably, SC8 contained the highest amounts of gomisin J (0.362 mg/g DW) and schisanhenol A (0.383 mg/g DW). These results demonstrate that the lignan profiles of SCE are highly region-dependent, with SC1 and SC5 being superior sources of multiple bioactive lignans [40].

### 3.3. Potential Geographical Distribution of Wild Schisandra chinensis in the Changbai Mountain Region

The MaxEnt model has emerged as a primary tool for predicting the potential distribution of plant and animal species on a global scale [41,42]. The AUC value is a critical metric for evaluating the accuracy of MaxEnt models [43]. Model performance was assessed by 10-fold cross-validation. The mean test AUC across the ten replicates was 0.8101 (SD = 0.0907). The cross-validated test AUC indicates that the model has reasonably good predictive ability without severe overfitting.

#### 3.3.1. Analysis of the Importance of Environmental Variables

Contribution rates can provide insights into the significance of each environmental variable in the construction of models. In this MaxEnt model prediction, we selected 13 environmental variables from WorldClim, including elevation, aspect, and temperature [44]. As shown in Table 4, the mean contribution rates of environmental variables over 10 cross-validation replicates indicated that bio13 (precipitation of the wettest month), bio16 (precipitation of the wettest quarter), bio1 (annual mean temperature), and t_gravel (gravel content of the upper soil layer) were the most influential factors, with average contributions of 42.16%, 28.61%, 7.88%, and 7.48%, respectively. Response curves for the four most influential variables were extracted from the MaxEnt output (Figure S3) This suggests that precipitation (particularly during the wettest month and wettest quarter), annual mean temperature, and upper-soil gravel content are crucial environmental factors influencing the distribution of *Schisandra chinensis*.

#### 3.3.2. *Schisandra chinensis* and Appropriate Area Division

**Figure 3:**
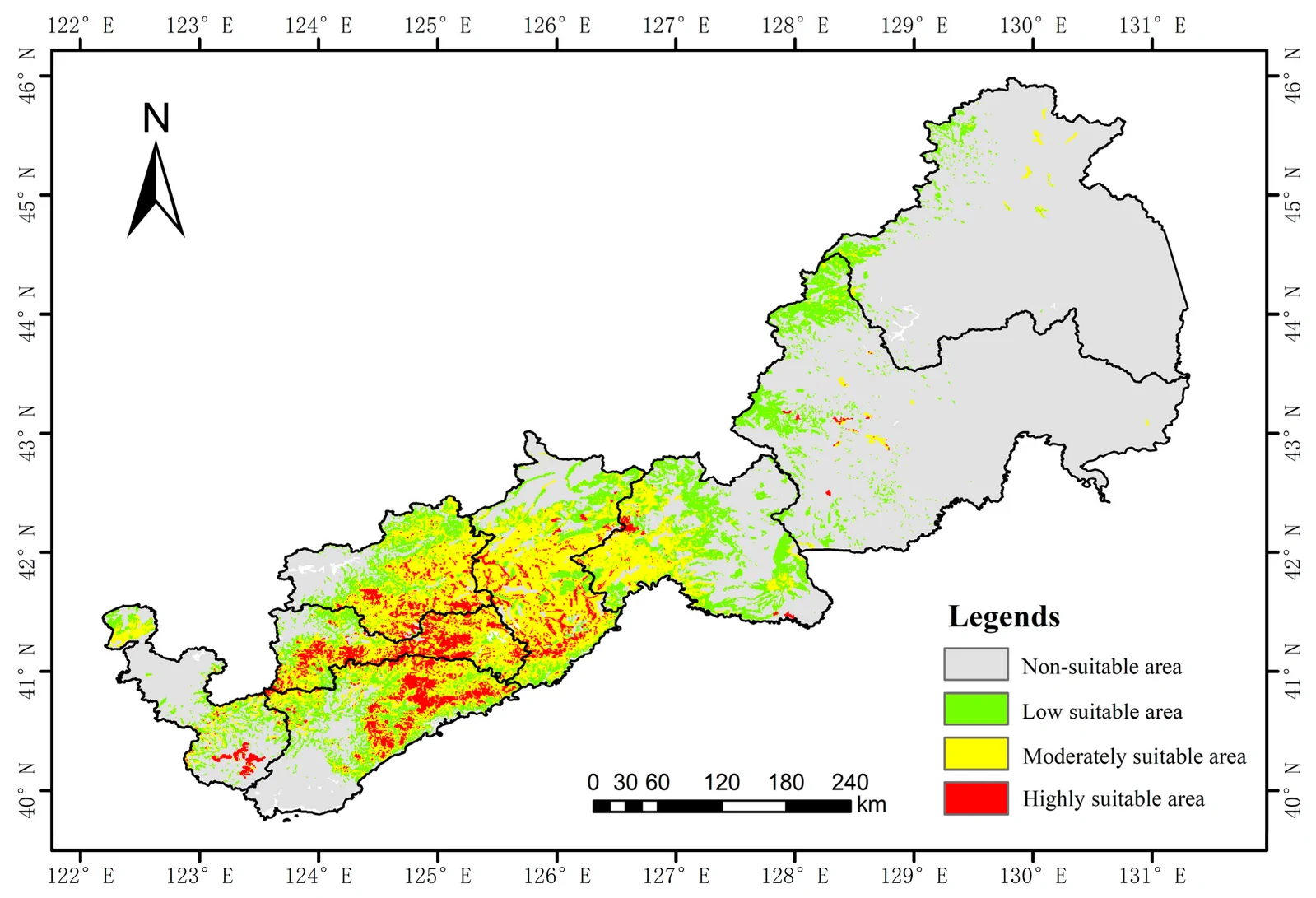
Potential distribution map of wild *Schisandra chinensis* in Changbai Mountain Region. Climate variables were sourced from the WorldClim database (http://www.worldclim.org/, accessed on 10 September 2025).

The MaxEnt analysis results (Figure 3) indicate that the potential distribution areas of *Schisandra chinensis* can be categorized into four levels based on the suitability index *p*-value, ranging from low to high: non-suitable, marginally suitable, moderately suitable, and highly suitable. The total area of suitable habitats (*p* ≥ 0.2) is 126,220.32 km^2^. Among these areas, the marginally suitable region covers 43,984.38 km^2^, accounting for 34.85% of the total area within the three northeastern provinces; the moderately suitable area spans 57,067.86 km^2^, representing 45.21% of the national total; and the highly suitable area comprises 25,168.08 km^2^, which constitutes 19.94%. Wild *Schisandra chinensis* establishes distinct suitable habitat circles in Kuandian Manchu Autonomous County of Dandong, Huanren Manchu Autonomous County of Benxi, Qingyuan and Xinbin Manchu Autonomous County of Fushun, and Tonghua County of Tonghua.

### 3.4. Physicochemical Characteristics of Rhizosphere Soil of S. chinensis

The soil properties of the eight sampling sites (SC1–SC8) varied markedly (Table 5). Soil pH ranged from 5.24 (SC4) to 7.53 (SC3), with most sites being acidic to weakly acidic, suitable for wild *Schisandra chinensis*. Soil organic matter (SOM) exhibited considerable variation: SC7 had the highest value (196.92 g/kg), followed by SC2 (175.88 g/kg) and SC5 (132.41 g/kg), while SC3 showed the lowest (16.36 g/kg). Total nitrogen (TN) ranged from 0.80 g/kg (SC3) to 10.16 g/kg (SC2), and available phosphorus (AP) varied between 3.69 mg/kg (SC3) and 22.19 mg/kg (SC7). Notably, SC2 and SC7 presented relatively higher AP levels (22.18 and 22.19 mg/kg, respectively), and SC8 also showed a moderately high AP content (20.77 mg/kg). These SOM and AP values are generally consistent with previous reports on wild *S. chinensis* rhizosphere soils, which are often rich in organic matter and available phosphorus due to forest humus accumulation [45,46]. For instance, studies have shown that wild *S. chinensis* in Northeast China frequently grows in soils with SOM exceeding 30% (300 g/kg) and AP ranging from 30 to over 100 mg/kg. In comparison, our measured SOM (maximum 196.92 g/kg) and AP (maximum 22.19 mg/kg) are lower than those reported thresholds, suggesting that the sampled sites represent moderately fertile natural forest soils. Nevertheless, the distinct lignan profiles observed in SC2, SC5, SC7, and SC8 may still be influenced by site-specific soil properties such as pH, TN, and AP.

### 3.5. Evaluation of the In Vitro Biological Activity of SCE in Different Regions

#### 3.5.1. Evaluation of In Vitro Antioxidant Activity of SCE in Different Regions

**Figure 4:**
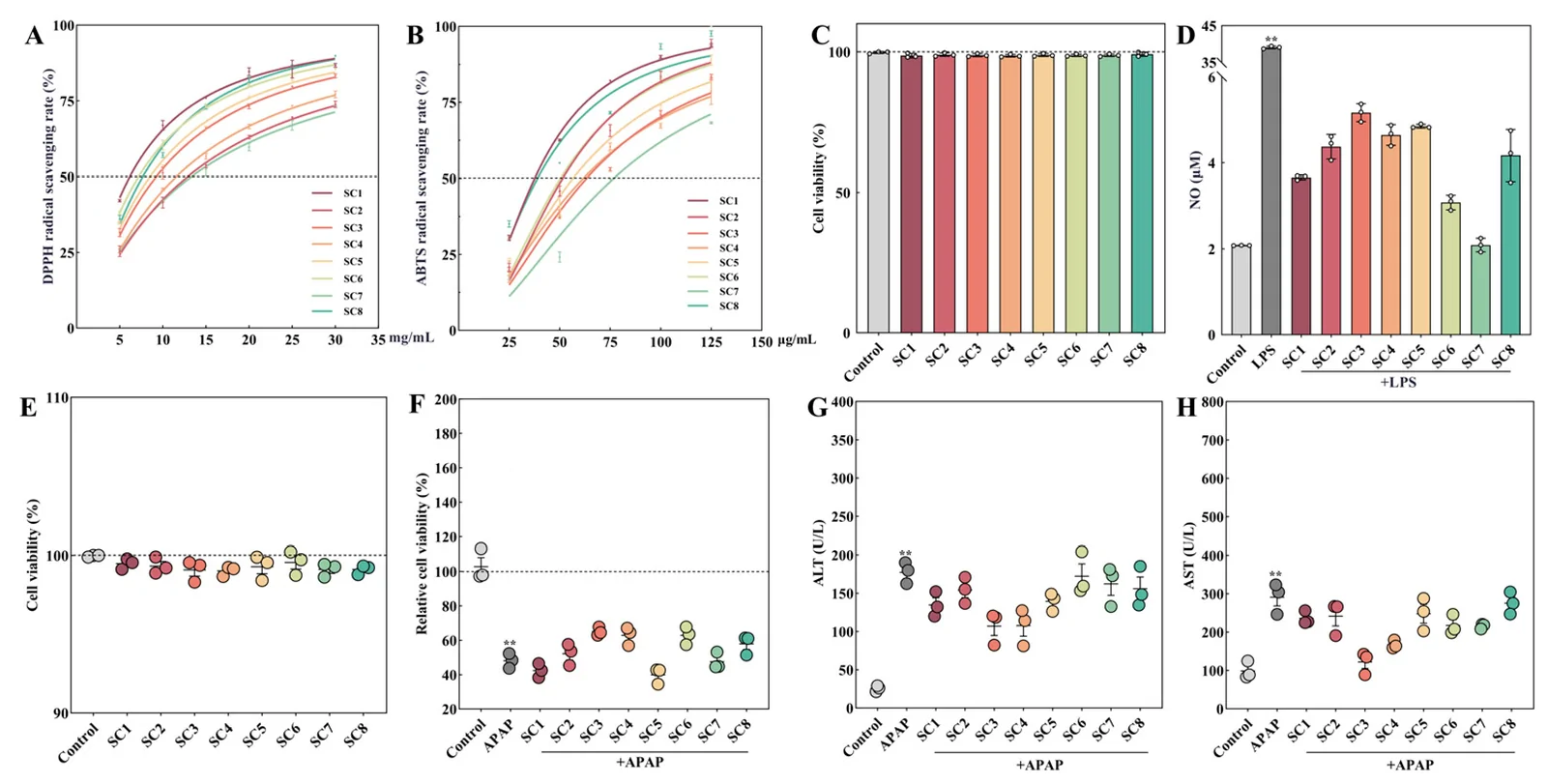
Comparison of SCE biological activity in different regions. (**A**) SCE’s scavenging activity against DPPH, (**B**) SCE’s scavenging activity against ABTS, (**C**) effects of SCE from different regions on the viability of RAW264.7 cells, (**D**) anti-inflammatory activity of SCE from different regions, (**E**) effects of SCE from different regions on the viability of AML12 cells, (**F**) hepatoprotective activity of SCE from different regions, (**G**) effects of SCE from different regions on ALT in the supernatant of AML12 cells, (**H**) effects of SCE from different regions on AST in the supernatant of AML12 cells. Results are presented as mean ± standard deviation, and each sample was tested three times.

Scavenging of reactive oxygen species (ROS) and alleviation of oxidative stress are key mechanisms through which many medicinal plants exert their pharmacological effects, including anti-aging effects, anti-inflammatory properties, and the prevention of metabolic diseases [47]. The antioxidant activity of *Schisandra chinensis* has been confirmed [23,27,48]. This study evaluated the antioxidant activity of *Schisandra chinensis* extract (SCE) from eight regions using free radical scavenging assays, specifically DPPH· and ABTS+. The DPPH method primarily reflects the ability of lipid-soluble components in extracts to neutralize free radicals through a hydrogen atom transfer mechanism, whereas the ABTS method is sensitive to both water-soluble and lipid-soluble antioxidants, with its reaction mechanism primarily involving electron transfer [49]. The combination of these two methods allows for a more comprehensive assessment of the antioxidant potential of samples under varying polarity environments and action mechanisms. The concentration-dependent scavenging activity of SCE was observed in both DPPH and ABTS assays (Figure 4A,B). For DPPH scavenging, SC1 exhibited the strongest trend (IC_50_ = 6.177 mg/mL) and SC7 the weakest (IC_50_ = 13.330 mg/mL); for ABTS scavenging, SC1 again showed the strongest trend (IC_50_ = 0.03765 mg/mL) and SC7 the weakest (IC_50_ = 0.07678 mg/mL) (Table 6). SC8 also demonstrated potent activity in both assays, ranking closely behind SC1. This study, utilizing two complementary antioxidant evaluation models (DPPH and ABTS), found that SC1 extracts consistently demonstrated the strongest activity, whereas SC7 exhibited the weakest performance (Table 6).

#### 3.5.2. Evaluation of Anti-Inflammatory Activity on RWA264.7 Cells

Nitric oxide (NO) is a crucial mediator of inflammatory responses, and thus, the inhibition of NO production can serve as a measure for evaluating anti-inflammatory effects [50]. *S. chinensis* lignans demonstrate their anti-inflammatory properties by promoting the release of cyclooxygenase-2 (COX-2) while simultaneously inhibiting nitric oxide synthase (NOS) to mitigate NO generation [51,52,53]. This study assessed the anti-inflammatory capacity by comparing the inhibitory effects of *Schisandra chinensis* extract (SCE) from various regions on NO production in lipopolysaccharide (LPS)-activated RAW264.7 cells. The impact of SCE from different regions on cell viability was evaluated using the CCK-8 assay. Cytotoxicity test results indicated that SCE from all regions exhibited no cytotoxicity at concentrations below 200 µg/mL (Figure 4C). As illustrated in Figure 4D, a highly significant difference was noted between the LPS group and the blank control (*p* < 0.01), Comparative analysis indicated that SC7 exhibited the most pronounced anti-inflammatory effect, followed by SC6 and SC1.

#### 3.5.3. Evaluation of Hepatoprotective Activity on AML12 Cells

Acetaminophen (APAP)-induced liver injury is a prevalent adverse drug reaction [25]. The hepatoprotective activity of *S. chinensis* extract and dibenzocyclooctadiene lignans is particularly noteworthy. Key compounds, including schisandrin A, schisandrin B, schisantherin A, and gomisin A, play a crucial role in promoting hepatocyte proliferation, enhancing hepatic blood flow, and reducing oxidative damage [13,52,54]. To assess the hepatoprotective effects of *S. chinensis* extract (SCE) from various regions, we established an APAP-induced AML12 cell injury model. The impact of SCE from different regions on cell viability was evaluated using the CCK-8 method. Cytotoxicity results indicated that SCE from all regions exhibited no cytotoxic effects at concentrations below 100 µg/mL (Figure 4E). Employing 100 µg/mL SCE to validate hepatoprotective effects revealed highly significant differences between the model and control groups (*p* < 0.01). SC3, SC4 and SC6 displayed relatively potent cytoprotective effects against induced damage (Figure 4F). Samples SC3 and SC4 exhibited relatively stronger inhibitory effects on the elevation of ALT and AST activities, while SC6 and SC7 also displayed obvious inhibitory tendencies against ALT and AST levels (Figure 4G,H). A comprehensive evaluation indicated that the hepatoprotective effect of SCE was optimal in the SC3 and SC4 regions.

#### 3.5.4. Evaluation of SCE Enzyme Inhibitory Activity in Different Regions

The development of enzyme inhibitors is an effective strategy for assessing functional foods [55], with α-amylase, α-glucosidase, and cholinesterase identified as the most frequently validated targets. These enzymes are closely linked to the onset and progression of chronic metabolic and neurodegenerative diseases, including diabetes, obesity, hyperlipidemia, and Alzheimer’s disease [56,57]. Such methods have been applied to grape pomace extract, *Althaea officinalis* L., and goji (*Lycium barbarum* L.) [33,58,59]. Furthermore, our findings indicate that SCE samples from different regions exhibit varying enzyme inhibitory activities. As illustrated in Table 6, SC6 showed the lowest IC50 value (4.989 mg/mL), suggesting the strongest trend in α-amylase inhibition among the tested samples, whereas SC8 exhibited the highest IC50 (7.121 mg/mL). For α-glucosidase inhibition, SC8 exhibited the strongest trend (IC_50_ = 4.880 mg/mL) and SC6 the weakest (IC_50_ = 7.460 mg/mL); for acetylcholinesterase inhibition, SC7 showed the strongest trend (IC_50_ = 0.608 mg/mL) and SC3 the weakest (IC_50_ = 0.930 mg/mL). SCEs from various regions demonstrate significantly different potencies and selectivities in hypoglycemic, lipid-lowering, and cholinesterase inhibitory activities. Regarding α-amylase inhibition, SC6 exhibited the strongest activity, while SC8 showed the weakest. In terms of α-glucosidase inhibition, SC8 displays the highest activity, whereas SC6 is the least active. For acetylcholinesterase inhibition, SC7 shows the most potent activity, while SC3 has the weakest.

### 3.6. Correlation Analysis of Environmental Factors with Lignans and Bioactivities

**Figure 5:**
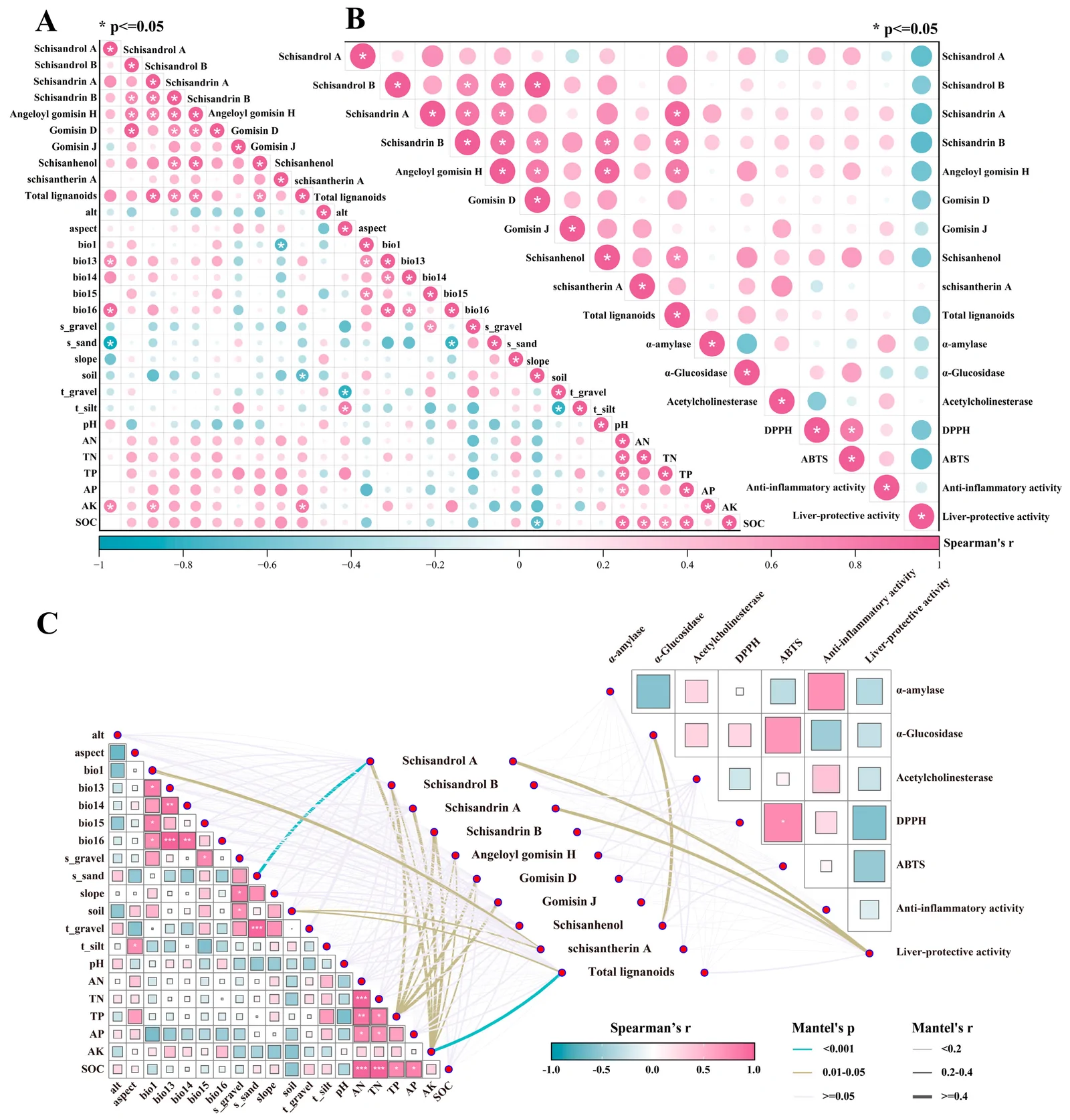
Correlation analysis of environmental factors, lignan content, and bioactivity. (**A**) Correlation heatmap between environmental/soil properties and individual lignans. (**B**) Correlation heatmap between bioactivities and individual lignans. (**C**) Partial Mantel tests (Euclidean distance, 9999 permutations) assessing the relationships between distance matrices. Line width and color intensity represent the Mantel r statistic. Given the limited sample size (*n* = 8 composite samples) and the exploratory nature of this study, the Mantel test results (environmental factors: r = 0.182, *p* = 0.229; biological factors: r = 0.053, *p* = 0.385) should be interpreted as indicating potential association trends rather than confirmed relationships, and require validation with independent biological replicates.

The growth environment is closely associated with fruit development and secondary metabolite profiles in plants, with soil properties and climatic factors playing important roles [60]. In *Schisandra chinensis*, habitat conditions and soil characteristics are closely associated with lignan accumulation patterns. To explore these associations, we analyzed correlations between lignan content and environmental factors (Figure 5A). Annual mean temperature (Bio1), precipitation (Bio13, Bio16), and soil sand content (s_sand) showed relatively strong associations with lignan variation. Consistent with Hu et al., Bio1, Bio13, and Bio16 are also known to influence the geographic distribution of *S. chinensis* [51]. Temperature and moisture are critical for plant growth, especially during fruit maturation (July–October) [61]. In our correlation analysis, annual mean temperature was negatively correlated with most lignans, with the most notable correlation observed for schisanhenol (*p* < 0.05). Conversely, precipitation showed positive correlations, particularly with schisandrol A (*p* < 0.05). Soil sand content was negatively correlated with most lignans, again most notably with schisandrol A (*p* < 0.05).

Soils in the study area were weakly acidic (pH 5.24–7.53), and lignan content was negatively correlated with pH (Figure 5A). This negative correlation suggests that weakly acidic conditions may be associated with enhanced lignan accumulation, potentially through improved solubility and uptake of micronutrients (e.g., Fe^3+^, Mn^2+^), which serve as cofactors for phenylalanine ammonia-lyase (PAL) and cinnamate 4-hydroxylase (C_4_H) in the phenylpropanoid pathway [62,63]. Thus, weakly acidic soil may be beneficial for lignan accumulation. Lignan content also showed positive correlations with soil organic carbon, total nitrogen, total phosphorus, and alkali-hydrolyzable nitrogen. Notably, schisandrol A, schisandrin A, and schisantherin A exhibited positive correlations with available potassium (*p* < 0.05) (Figure 5A). The positive correlations of available P and K with specific lignans (e.g., schisandrol B, gomisin J) suggest that these macronutrients are associated with the accumulation of these lignans, consistent with their known roles in plant secondary metabolism [64,65]. Overall, moderate temperature, adequate precipitation, and slightly acidic, fertile soils showed positive associations with lignan accumulation, whereas altitude exhibited limited direct correlation.

Regarding bioactivity (Figure 5B), lignans such as schisandrol A, angeloyl gomisin H, and schisanhenol showed strong positive correlations with ABTS and DPPH radical scavenging capacities. Total lignan content was positively correlated with α-glucosidase and α-amylase inhibitory activities, and schisandrol A, angeloyl gomisin H, and schisanhenol exhibited the strongest correlations with α-glucosidase inhibition, suggesting potential relevance to glucose metabolism regulation. Hepatoprotective activity was positively correlated with most lignans, supporting the traditional use of *S. chinensis* as a liver-protective agent. Acetylcholinesterase inhibition showed mixed correlation patterns: negative with schisandrol A, but positive with schisantherin A and gomisin J, indicating component-specific effects. Anti-inflammatory activity was more weakly correlated with lignans, suggesting that this activity may involve synergistic actions with other metabolites.

Previous studies link lignan and bioactivity differences between *Schisandra sphenanthera* Rehder & E.H.Wilson and *S. chinensis* (Turcz.) Baill. to habitat variation [66,67]. Our Mantel test (Figure 5C), together with Spearman‘s correlation analysis (Figure 5A,B), revealed several suggestive association patterns. Although the overall correlation between the environmental matrix and the lignan matrix did not reach statistical significance (Mantel r = 0.182, *p* = 0.229), climatic factors, particularly temperature (Bio1) and precipitation (Bio13, Bio16), showed relatively stronger correlations with lignan profiles, whereas soil properties—especially pH, available phosphorus, and available potassium—exhibited more pronounced associations with individual lignan accumulation. Specifically, warm and humid conditions, together with weakly acidic soils (pH 5.2–6.5) and higher available P and K levels, were positively associated with the accumulation of key lignans (schisandrol A, schisandrol B, schisandrin B, and schisantherin A). These lignans, in turn, showed positive correlations with antioxidant, hepatoprotective, and hypoglycemic activities (Figure 5B). From a practical perspective, these correlational patterns suggest that selecting warm, humid, fertile habitats, or applying agronomic measures such as organic fertilization and pH adjustment, may be beneficial for enhancing target lignan content. For instance, improving soil P and K availability was associated with elevated levels of schisandrol A, schisandrol B, schisantherin A, schisantherin B, angeloyl gomisin H, gomisin J, and gomisin D, which in turn showed associations with enhanced liver-protective activity in our in vitro assays, supporting the potential development of region-specific functional foods. However, given the observational and exploratory nature of this study, these proposed associations should be interpreted with caution and require further validation through controlled experiments or mechanistic studies.

## 4. Limitations

This study has several limitations that should be considered when interpreting the findings. First, only one composite sample (pooled from 20 plants) was obtained per location due to resource scarcity and restricted access to nature reserves [16,20,21]. Technical replicates cannot substitute for biological replicates; thus, all statistical comparisons are exploratory and require validation with independent sampling. Second, the PCA, OPLS-DA, and Mantel tests were based on a limited sample size (*n* = 8), and the multivariate results should be interpreted as hypothesis-generating. Third, absolute matrix effects and spike recovery were not evaluated because the study was designed for relative comparison among origins using an identical processing protocol; future absolute-quantification studies should include these validations. Fourth, bioactivities were assessed only in vitro, and the identified environment–metabolite associations are correlative; in vivo studies and mechanistic experiments are needed to establish causation. Fifth, sampling was confined to a single season (2024), and the MaxEnt model relied on only 20 occurrence points [30,31]; the habitat suitability map (Figure 3) is therefore exploratory. Despite these caveats, the integrated dataset provides a useful preliminary framework for understanding regional variation in the functional quality of wild *S. chinensis*.

## 5. Conclusions

This study exploratorily compared the metabolomic profiles, lignan accumulation, and in vitro bioactivities of wild *Schisandra chinensis* from eight geographical origins in the Changbai Mountain region, and identified key environmental factors showing associations with functional quality. Eighteen differential metabolites, including nine lignans, were identified, with total lignan content ranging from 7.71 to 13.72 mg/g DW across sites. Bioactivities varied significantly by origin: SC1 (Liaoning) exhibited the most favorable antioxidant trend; SC7 (Jilin) showed relatively strong anti-inflammatory and acetylcholinesterase inhibition; SC6 (Jilin) and SC8 (Heilongjiang) displayed relatively strong α-amylase and α-glucosidase inhibition, respectively; and SC3/SC4 (Liaoning) had the best hepatoprotective effects. Environmental correlation analysis revealed that a warm–humid climate, weakly acidic soil (pH 5.2–6.5), and higher available phosphorus and potassium were associated with higher lignan levels, suggesting that these factors may play a role in lignan accumulation, although the observational nature of this study precludes causal inference. These findings provide actionable guidance for origin authentication (e.g., chemical markers for distinguishing regions), soil amendment (e.g., optimizing pH and P/K fertilization), and region-specific harvesting (e.g., selecting SC7 for anti-inflammatory products). Our findings suggest the potential for region-specific harvesting strategies and soil management practices to optimize bioactive lignan accumulation. Given the screening-oriented nature of this study, the mechanisms underlying the observed region-specific bioactivities remain to be elucidated. Nonetheless, our results provide a valuable reference for the targeted development of region-specific functional foods and for prioritizing candidate regions for subsequent mechanistic investigations.

## Figures and Tables

**Table 1:** Sample information.

Sample ID	Province	Region	Longitude/E	Latitude/N	Altitude
SC1	Liaoning (A)	Kuandian County, Dandong City	124.4958	40.4375	334
SC2		Huanren County, Benxi City	125.8458	41.7458	450
SC3		Xiuyan Manchu Autonomous County, Anshan City	123.1041	40.2791	242
SC4		Xinbin County, Fushun City	124.7458	41.8791	560
SC5	Jilin (B)	Tonghua County, Tonghua City	125.3291	41.3208	548
SC6		Baishan County, Fusong City	126.5625	41.9708	545
SC7		Anju County, Yanbian Korean Autonomous Prefecture	128.4791	43.1208	800
SC8	Heilongjiang (C)	Beihai Town, Ning’an City, Mudanjiang City	129.6291	45.6875	217

**Table 2:** Identification of the chemical components of SCE.

No.	Rt (min)	Ion Mode	MS/MS	Theoretical *m*/*z*	Measured *m*/*z*	Error ppm	Formula	Proposed Molecule
1	1.36	[M + H]^+^	380.09, 290.08 233.06	308.09088	308.09080	−0.26	C_10_H_17_N_3_O_6_S	Glutathione
2	6.25	[M − H]^−^	59.01, 71.01 89.03	383.11952	383.11960	0.21	C_14_H_24_O_12_	Acetyl-maltose
3	9.27	[M + H]^+^	165.02, 205.01 138.03	341.12422	341.12409	−0.38	C_16_H_22_O_8_	Coniferin
4	10.12	[M + H]^+^	199.08, 227.09 111.01	361.09098	361.09041	−1.58	C_18_H_16_O_8_	Rosmarinic acid
5	11.15	[M + H]^+^	287.06, 153.02 121.03	449.10548	449.10550	0.04	C_21_H_20_O_11_	Kaempferol-7-O-glucoside
6	11.82	[M − H]^−^	165.02, 137.02 166.02	301.03522	301.03532	0.33	C_15_H_10_O_7_	Quercetin
7	12.10	[M + H]^+^	300.03, 301.03 271.03	435.09198	435.09190	−0.18	C_20_H_18_O_11_	Avicularin
8	12.43	[M + H − H_2_O]^+^	119.09, 105.07 175.15	175.14811	175.14810	−0.06	C_13_H_20_O	β-ionone
9	15.69	[M − H]^−^	153.02, 131.05 257.08	255.06602	255.06606	0.16	C_15_H_12_O_4_	Pinocembrin
10	16.11	[M + H]^+^	287.09, 227.07 325.14	389.19528	389.19522	−0.15	C_22_H_28_O_6_	Gomisin J
11	17.33	[M + H]^+^	515.18, 371.15 340.13	417.19058	417.19071	0.31	C_28_H_34_O_9_	Schisandrol B
12	17.87	[M − H]^−^	425.20, 471.20 161.06	471.20088	471.20040	−1.02	C_26_H_30_O_8_	Limonoate D-ring-lactone
13	17.91	[M + H]^+^	287.06, 137.02 213.06	595.16538	595.16532	−0.10	C_27_H_30_O_15_	Cyanidin 3-O-rutinoside
14	19.23	[M + H]^+^	369.17, 401.20 370.18	401.19528	401.19537	+0.22	C_23_H_28_O_6_	Schisandrin B
15	19.96	[M + H]^+^	223.09, 385.15 386.15	385.16418	385.16414	−0.10	C_22_H_24_O_6_	Schisandrin C
16	20.03	[M + H]^+^	95.09, 163.15 189.16	457.36758	457.36700	−1.27	C_30_H_48_O_3_	Ursolic acid
17	22.73	[M + H]^+^	465.33, 466.33 433.37	443.38838	443.38820	−0.41	C_30_H_50_O_2_	Betulin
18	24.49	[M + Na]^+^	367.39, 71.01 187.95	391.35422	391.35410	−0.31	C_24_H_48_O_2_	Lignoceric acid

**Table 3:** Concentration of Lignans in *Schisandra chinensis* from Different Origins (mean ± SD, mg/g DW).

Sample ID	Schisandrol A	Schisandrol B	Schisandrin A	Schisandrin B	Angeloylgomisin H	Gomisin J	Gomisin D	Schisanhenol	Schisantherin A	Total Lignans
SC1	8.060 ± 0.028	0.317 ± 0.009	0.732 ± 0.012	1.827 ± 0.015	1.585 ± 0.016	0.137 ± 0.006	0.317 ± 0.009	0.085 ± 0.005	0.121 ± 0.006	13.713 ± 0.080
SC2	5.952 ± 0.020	0.255 ± 0.007	0.607 ± 0.015	0.966 ± 0.014	0.776 ± 0.011	0.121 ± 0.007	0.255 ± 0.007	0.070 ± 0.006	0.130 ± 0.008	9.563 ± 0.008
SC3	5.856 ± 0.026	0.220 ± 0.007	0.344 ± 0.011	0.755 ± 0.013	0.687 ± 0.012	0.066 ± 0.007	0.220 ± 0.007	0.038 ± 0.004	0.126 ± 0.007	8.627 ± 0.007
SC4	4.178 ± 0.019	0.283 ± 0.008	0.309 ± 0.006	0.829 ± 0.009	0.757 ± 0.011	0.164 ± 0.007	0.283 ± 0.008	0.046 ± 0.004	0.091 ± 0.007	7.709 ± 0.021
SC5	6.255 ± 0.026	0.369 ± 0.009	0.707 ± 0.013	2.382 ± 0.014	1.340 ± 0.011	0.323 ± 0.011	0.369 ± 0.009	0.083 ± 0.006	0.136 ± 0.008	13.115 ± 0.016
SC6	5.029 ± 0.025	0.248 ± 0.008	0.477 ± 0.008	1.025 ± 0.018	0.710 ± 0.012	0.176 ± 0.009	0.248 ± 0.008	0.055 ± 0.005	0.112 ± 0.007	8.607 ± 0.073
SC7	4.928 ± 0.024	0.278 ± 0.008	0.668 ± 0.009	1.655 ± 0.016	0.858 ± 0.012	0.307 ± 0.007	0.278 ± 0.008	0.079 ± 0.006	0.166 ± 0.007	10.022 ± 0.014
SC8	5.010 ± 0.026	0.262 ± 0.007	0.465 ± 0.012	1.059 ± 0.014	0.992 ± 0.012	0.362 ± 0.009	0.262 ± 0.007	0.086 ± 0.006	0.383 ± 0.009	9.855 ± 0.031

Note: SD represents technical precision rather than biological variation.

**Table 4:** Mean contribution rates (%) of environmental variables based on 10-fold cross-validation (10 replicates) in the MaxEnt model.

Environment Variables	Variable Description	Contribution (Mean ± SD, %)
Alt	Altitude	1.41 ± 1.19
Aspect	Aspect	1.46 ± 1.01
bio1	Annual average temperature	7.88 ± 3.47
bio13	The precipitation in the wettest month	42.16 ± 12.57
bio14	The precipitation in the driest month	0.02 ± 0.06
bio15	Seasonal variation of precipitation	1.48 ± 0.96
bio16	The precipitation during the wettest season	28.61 ± 12.51
s_gravel	The content of gravel in the lower layer of soil	1.40 ± 1.80
s_sand	The amount of sand in the lower layer of soil	0.27 ± 0.18
slope	Slope	1.42 ± 0.88
soil	Soil type	3.16 ± 1.08
t_gravel	The content of gravel in the upper layer of soil	7.48 ± 2.49
t_silt	The content of silt in the lower layer of soil	3.25 ± 1.55
Mean test AUC		0.8101 ± 0.0907

Note: Data are presented as mean ± SD calculated from 10 replicate runs. Test AUC is the area under the ROC curve averaged over the 10 replicates.

**Table 5:** Content of Soil Factors in Different Regions (mean ± SD).

Sample ID	pH	AN (mg/kg)	TN (g/kg)	TP (g/kg)	AP (mg/kg)	AK (mg/kg)	SOM (g/kg)
SC1	5.55 ± 0.04 ab	191.02 ± 9.03 ab	2.47 ± 0.04 ab	0.74 ± 0.08 ab	9.06 ± 0.08 ab	243.82 ± 6.45 ab	53.43 ± 1.69 ab
SC 2	6.45 ± 0.11 ab	558.57 ± 57.94 a	10.16 ± 0.44 b	1.04 ± 0.06 ab	22.18 ± 1.45 b	156.19 ± 4.19 ab	175.88 ± 6.52 ab
SC 3	7.53 ± 0.16 a	56.15 ± 9.67 b	0.80 ± 0.03 a	0.25 ± 0.03 b	3.69 ± 0.17 a	174.56 ± 7.42 ab	16.36 ± 1.38 a
SC 4	5.24 ± 0.08 b	198.16 ± 6.50 ab	2.69 ± 0.03 ab	0.84 ± 0.07 ab	5.41 ± 0.09 ab	125.13 ± 5.21 b	50.15 ± 0.49 ab
SC 5	6.15 ± 0.04 ab	516.90 ± 21.60 a	7.73 ± 0.35 ab	1.93 ± 0.05 a	8.59 ± 0.13 ab	296.29 ± 5.76 a	132.41 ± 3.47 ab
SC 6	6.15 ± 0.14 ab	145.88 ± 4.29 ab	1.67 ± 0.04 ab	0.74 ± 0.09 ab	5.72 ± 0.14 ab	126.10 ± 5.03 b	29.82 ± 0.33 ab
SC 7	5.59 ± 0.14 ab	418.29 ± 33.49 ab	7.57 ± 0.09 ab	1.02 ± 0.05 ab	22.19 ± 1.32 b	170.98 ± 1.89 ab	196.92 ± 3.78 b
SC 8	5.47 ± 0.1 ab	234.88 ± 27.80 ab	2.41 ± 0.07 ab	1.22 ± 0.24 ab	20.77 ± 0.91 ab	155.78 ± 0.82 ab	54.26 ± 0.98 ab

Note: Values are mean ± SD of three independent field replicates (*n* = 3) per site. Different lowercase letters within a column indicate significant differences among sites (*p* < 0.05). pH: pH; SOM: Soil Organic Matter; TN: Total Nitrogen; TP: Total Phosphorus; AN: Available Nitrogen; AP; Available Phosphorus; AK: Available Potassium.

**Table 6:** Antioxidant and Enzyme Inhibitory Capabilities of SCE in Different Regions.

Sample ID	IC_50_ mg/mL				
	α-Amylase	α-Glucosidase	Acetylcholinesterase	DPPH Free Radical	ABTS+ Free Radical
SC1	6.230	5.661	0.7433	6.177	0.03765
SC2	5.890	5.704	0.6225	12.890	0.05150
SC3	6.251	6.716	0.9295	9.243	0.06334
SC4	6.854	6.848	0.7415	11.570	0.06172
SC5	5.770	5.713	0.6521	8.527	0.05661
SC6	4.989	7.460	0.6667	7.197	0.05045
SC7	5.492	6.888	0.6080	13.330	0.07678
SC8	7.121	4.880	0.6322	7.678	0.03905
Control	5.896	1.667	0.1146	0.06176	0.00106

## Data Availability

The original contributions presented in this study are included in the article/Supplementary Materials. Further inquiries can be directed to the corresponding author.

## Supplementary Materials

The following supporting information can be downloaded at: https://www.mdpi.com/article/10.3390/foods15213941/s1, Figure S1. Permutation test (200 random permutations) for the OPLS-DA model; Figure S2. Standard and sample chromatograms; Figure S3. Response curves of the four most influential environmental variables for Schisandra chinensis based on MaxEnt single-variable models (_only). The y-axis represents the logistic output (habitat suitability), and the x-axis shows the range of each environmental variable; Table S1. Molecular weight detection parameters of each component; Table S2. Data Information of Wild Schisandra chinensis Samples in the Changbaishan Mountain Region; Table S3. Regression data for the analysis of 9 types of lignans using high-performance liquid chromatography; Table S4. Method validation for the 9 lignans of LC-MS.

## References

[1] De Castro I.C., Pequito D.C.T., Borghetti G., Yamaguchi A.A., de Brito G.A.P., Yamazaki R.K., Pôrto L.C.J., Coimbra T.M., Fernandes L.C., Fernandez R. (2023). Obesity-like metabolic effects of high-carbohydrate or high-fat diets consumption in metabolic and renal functions. Arch. Physiol. Biochem..

[2] Leidmaa E., Zimmer A., Stein V., Gellner A.K. (2025). Acute high-fat high-sugar diet rapidly increases blood-brain barrier permeability in mice. J. Nutr. Health Aging.

[3] Dama A., Shpati K., Daliu P., Dumur S., Gorica E., Santini A. (2024). Targeting Metabolic Diseases: The Role of Nutraceuticals in Modulating Oxidative Stress and Inflammation. Nutrients.

[4] Sharma V., Mehdi M.M. (2023). Oxidative stress, inflammation and hormesis: The role of dietary and lifestyle modifications on aging. Neurochem. Int..

[5] Golovinskaia O., Wang C.K. (2021). Review of Functional and Pharmacological Activities of Berries. Molecules.

[6] Ruxton C.H.S., Derbyshire E., Sievenpiper J.L. (2021). Pure 100% fruit juices—More than just a source of free sugars? A review of the evidence of their effect on risk of cardiovascular disease, type 2 diabetes and obesity. Nutr. Bull..

[7] Sorrenti V., Burò I., Consoli V., Vanella L. (2023). Recent Advances in Health Benefits of Bioactive Compounds from Food Wastes and By-Products: Biochemical Aspects. Int. J. Mol. Sci..

[8] Olas B. (2023). Cardioprotective Potential of Berries of *Schisandra chinensis* Turcz. (Baill.), Their Components and Food Products. Nutrients.

[9] Szopa A., Ekiert R., Ekiert H. (2017). Current knowledge of *Schisandra chinensis* (Turcz.) Baill. (China magnolia vine) as a medicinal plant species: A review on the bioactive components, pharmacological properties, analytical and biotechnological studies. Phytochem. Rev..

[10] Zhou F., Wang M., Ju J., Wang Y., Liu Z., Zhao X., Yan Y., Yan S., Luo X., Fang Y. (2019). Schisandrin A protects against cerebral ischemia-reperfusion injury by suppressing inflammation and oxidative stress and regulating the AMPK/Nrf2 pathway regulation. Am. J. Transl. Res..

[11] Korea Heritage Service Hwachae Soothes the Summer with Fruity Flavors. Korean Heritage. http://koreanheritage.kr/feature/view_m.jsp?articleNo=101.

[12] Yang H.J., Kim M.J., Lee H.Y., Jang D.J., Park S. (2025). Scientific validation of traditionally used omija (*Schisandra chinensis*) extract mixture for postprandial glucose regulation. J. Ethn. Foods.

[13] Panossian A., Wikman G. (2008). Pharmacology of *Schisandra chinensis* Bail.: An overview of Russian research and uses in medicine. J. Ethnopharmacol..

[14] Jang S., Yuan C. (2021). *Schisandra chinensis*: A comprehensive review on its phytochemicals and biological activities. Arab. J. Chem..

[15] Zhou Y., Men L., Sun Y., Wei M., Fan X. (2021). Pharmacodynamic effects and molecular mechanisms of lignans from *Schisandra chinensis* Turcz. (Baill.), a current review. Eur. J. Pharmacol..

[16] Jia E., Dong J., Ma P. (2022). A new industrial model: The utilization of the traditional Chinese herb *Schisandra chinensis* (Turcz.) Baill. from soil to plate. Ind. Crops Prod..

[17] Wadood S.A., Nie J., Song Y., Li C., Rogers K.M., Khan W.A., Khan A., Xiao J., Liu H., Yuan Y. (2025). Authentication of edible herbal materials and food products using mass spectrometry based metabolites and inorganic constituents. Food Chem..

[18] Poudeyal M.R., Meilby H., Hart R. (2024). Sustainable harvest of a threatened medicinal herb: Empirical evidence for spatially and temporally specific management of *Neopicrorhiza scrophulariiflora*. Perspect. Plant Ecol. Evol. Syst..

[19] He D., Pei X., Liu B., Li J., Dong J., Efferth T., Ma P. (2024). Lignan contents of *Schisandra chinensis* (Turcz.) Baill. from different origins—A new model for evaluating the content of prominent components of Chinese herbs. Phytomedicine.

[20] Bai F., Sang W., Li G., Liu R., Chen L., Wang K. (2008). Long-term protection effects of National Reserve to forest vegetation in 4 decades: Biodiversity change analysis of major forest types in Changbai Mountain Nature Reserve, China. Sci. China Ser. C Life Sci..

[21] Ye J.W., Zhang Z.K., Wang H.F., Bao L., Ge J.P. (2019). Phylogeography of *Schisandra chinensis* (Magnoliaceae) Reveal Multiple Refugia with Ample Gene Flow in Northeast China. Front. Plant Sci..

[22] (1999). Field Sampling and Preparation of Forest Soil Samples.

[23] Kopustinskiene D.M., Bernatoniene J. (2021). Antioxidant Effects of *Schisandra chinensis* Fruits and Their Active Constituents. Antioxidants.

[24] Xie G.R., Chen H.J. (2021). Comprehensive Betalain Profiling of Djulis (*Chenopodium formosanum*) Cultivars Using HPLC-Q-Orbitrap High-Resolution Mass Spectrometry. J. Agric. Food Chem..

[25] Wang X., Su Z., Li X., Chen J., Li G., Shan Y., Pan Z., Fu F. (2025). Targeted/untargeted metabolomics and antioxidant properties distinguish Citrus reticulata ‘Chachi’ from Citrus reticulata Blanco. Food Chem..

[26] Ahn S.J., Kim H.J., Lee A., Min S.S., In S., Kim E. (2020). Determination of 12 herbal compounds for estimating the presence of Angelica Gigas Root, Cornus Fruit, Licorice Root, Pueraria Root, and *Schisandra* Fruit in foods by LC-MS/MS. Food Addit. Contam. Part A Chem. Anal. Control Expo. Risk Assess..

[27] Wang X.T., Yu J.H., Li W., Wang C., Li H., Ju W., Chen J., Sun J. (2018). Characteristics and antioxidant activity of lignans in *Schisandra chinensis* and *Schisandra sphenanthera* from different locations. Chem. Biodivers..

[28] Guo Y., Wei H., Lu C., Gao B., Gu W. (2016). Predictions of potential geographical distribution and quality of *Schisandra sphenanthera* under climate change. PeerJ.

[29] Phillips S.J., Dudík M. (2008). Modeling of species distributions with Maxent: New extensions and a comprehensive evaluation. Ecography.

[30] Lobo J.M., Jiménez-Valverde A., Real R. (2008). AUC: A misleading measure of the performance of predictive distribution models. Glob. Ecol. Biogeogr..

[31] Wisz M.S., Hijmans R.J., Li J., Peterson A.T., Graham C.H., Guisan A. (2008). Effects of sample size on the performance of species distribution models. Divers. Distrib..

[32] Wang Y., Lu W., Li J., Yang Y., Fan S., Wang Y., Qin H., Shu N., Zhang B., Li C. (2025). Rhizosphere Microbiota and Soil Nutrients Shape Fruit Lignan Composition of *Schisandra chinensis* Across Temperate Cultivation Sites in Northeast and Northwest China. Life.

[33] Marinaccio L., Gentile G., Llorent-Martínez E.J., Zengin G., Masci D., Flamminii F., Stefanucci A., Mollica A. (2025). Valorization of grape pomace extracts against cranberry, elderberry, rose hip berry, goji berry and raisin extracts: Phytochemical profile and in vitro biological activity. Food Chem..

[34] Liu J., Mu X., Liang J., Zhang J., Qiang T., Li H., Li B., Liu H., Zhang B. (2022). Metabolic profiling on the analysis of different parts of *Schisandra chinensis* based on UPLC-QTOF-MS with comparative bioactivity assays. Front. Plant Sci..

[35] Zhou Y., Fan X., Jiao T., Li W., Chen P., Jiang Y., Sun J., Chen Y., Chen P., Guan L. (2021). SIRT6 as a key event linking P53 and NRF2 counteracts APAP-induced hepatotoxicity through inhibiting oxidative stress and promoting hepatocyte proliferation. Acta Pharm. Sin. B.

[36] R Core Team (2022). R: A Language and Environment for Statistical Computing.

[37] Lever J., Krzywinski M., Altman N. (2017). Points of significance: Principal component analysis. Nat. Methods.

[38] Trygg J., Holmes E., Lundstedt T. (2007). Chemometrics in metabonomics. J. Proteome Res..

[39] Brereton R.G. (2009). Chemometrics for Pattern Recognition.

[40] The State Pharmacopoeia Commission of China (2020). Pharmacopoeia of the People’s Republic of China, Part I.

[41] Araujo M.B., Luoto M. (2007). The importance of biotic interactions for modelling species distributions under climate change. Glob. Ecol. Biogeogr..

[42] Chen C.Y., Liu S.Y., Yan Y., Yin L., Di P., Liu H.M., Liu H.Z. (2020). Candidate genes involved in the biosynthesis of lignan in *Schisandra chinensis* fruit based on transcriptome and metabolomes analysis. Chin. J. Nat. Med..

[43] Qin X., Li M. (2023). Predicting the Potential Distribution of *Oxalis debilis* Kunth, an Invasive Species in China with a Maximum Entropy Model. Plants.

[44] Akyol A., Orucu O.K., Arslan E.S. (2023). Predicting of the current and future geographical distribution of *Laurus nobilis* L. under the effects of climate change. Environ. Monit. Assess..

[45] Cho J.Y. (2010). Characteristics of *Schizandra chinensis* Baillon Orchard Soils Located in Jangsu-gun, Jeollabuk-do. Korean J. Soil Sci. Fertil..

[46] Yan B.Q., Luo Z.L., Hu L.L. (2013). Population Characteristics of Wild *Schisandra chinensis* in the Northeast of China and Its Relationship with Environmental Factors. Res. Environ. Sci..

[47] Limsuwan S., Awaeloh N., Na-Phatthalung P., Kaewmanee T., Chusri S. (2025). Exploring Antioxidant Properties of Standardized Extracts from Medicinal Plants Approved by the Thai FDA for Dietary Supplementation. Nutrients.

[48] Liu W., Chen X., Zhao Y., Shi H. (2025). Effects of green manuring on chemical characteristics and microecology of tobacco-growing soil in central Henan. BMC Microbiol..

[49] Munteanu I.G., Apetrei C. (2021). Analytical Methods Used in Determining Antioxidant Activity: A Review. Int. J. Mol. Sci..

[50] Xia G.Y., Yao T., Zhang B.Y., Li Y., Kang N., Cao S.J., Ding L.Q., Chen L.X., Qiu F. (2017). Withapubesides A-D: Natural inducible nitric oxide synthase (iNOS) inhibitors from *Physalis pubescens*. Org. Biomol. Chem..

[51] Hu L.L., Zhang H.Y., Qin L., Yan B.Q. (2012). Current distribution of *Schisandra chinensis* in China and its predicted responses to climate change. Chin. J. Appl. Ecol..

[52] Jiang Y., Fan X., Wang Y., Tan H., Chen P., Zeng H., Huang M., Bi H. (2015). Hepato-protective effects of six schisandra lignans on acetaminophen-induced liver injury are partially associated with the inhibition of CYP-mediated bioactivation. Chem. Biol. Interact..

[53] Waiwut P., Shin M.S., Yokoyama S., Saiki I., Sakurai H. (2012). Gomisin A enhances tumor necrosis factor-α-induced G1 cell cycle arrest via signal transducer and activator of transcription 1-mediated phosphorylation of retinoblastoma protein. Biol. Pharm. Bull..

[54] Thandavarayan R.A., Giridharan V.V., Arumugam S., Suzuki K., Ko K.M., Krishnamurthy P., Watanabe K., Konishi T. (2015). Schisandrin B prevents doxorubicin induced cardiac dysfunction by modulation of DNA damage, oxidative stress and inflammation through inhibition of MAPK/p53 signaling. PLoS ONE.

[55] Zengin G., Locatelli M., Carradori S., Mocan A.M., Aktumsek A. (2016). Total phenolics, flavonoids, condensed tannins content of eight Centaurea species and their broad inhibitory activities against cholinesterase, tyrosinase, α-amylase and α-glucosidase. Not. Bot. Horti Agrobot. Cluj-Napoca.

[56] Anshika R.K., Pandey L.S., Kumar S., Singh P., Pathak M., Jain S. (2022). Plant bioactive compounds and their mechanistic approaches in the treatment of diabetes: A review. Future J. Pharm. Sci..

[57] Zhang H., Peng Y., Zhuo L., Wang Y., Zeng G., Wang S., Long L., Li X., Wang Z. (2022). Recent advance on pleiotropic cholinesterase inhibitors bearing amyloid modulation efficacy. Eur. J. Med. Chem..

[58] Sicari V., Romeo R., Mincione A., Santacaterina S., Tundis R., Loizzo M.R. (2023). Ciabatta bread incorporating goji (*Lycium barbarum* L.): A new potential functional product with impact on human health. Foods.

[59] Xue T.T., Yang Y.G., Tang Z.S., Duan J.A., Song Z.X., Hu X.H., Yang H.D., Xu H.B. (2022). Evaluation of antioxidant, enzyme inhibition, nitric oxide production inhibitory activities and chemical profiles of the active extracts from the medicinal and edible plant: *Althaea officinalis* L.. Food Res. Int..

[60] Kandimalla R., Das M., Barge S.R., Sarma P.P., Koiri D.J., Devi A., Karki A.K., Kumar A., Devi R., Pal B.C. (2020). Variation in biosynthesis of an effective anticancer secondary metabolite, mahanine in *Murraya koenigii*, conditional on soil physicochemistry and weather suitability. Sci. Rep..

[61] Trisurat Y., Shrestha R.P., Kjelgren R. (2011). Plant species vulnerability to climate change in Peninsular Thailand. Appl. Geogr..

[62] Chen Y., Lu S., Mao L. (2021). Prediction of future changes in suitable distribution area for rare tree species of Dalbergia. J. Zhejiang A&F Univ..

[63] Zhang Y., Shi J., Liu L., Gao Z., Che J., Shao D., Liu Y.l. (2015). Bioconversion of Pinoresinol Diglucoside and Pinoresinol from Substrates in the Phenylpropanoid Pathway by Resting Cells of *Phomopsis* sp. XP-8. PLoS ONE.

[64] Deng Y., Lu S. (2017). Biosynthesis and regulation of phenylpropanoids in plants. Crit. Rev. Plant Sci..

[65] Liu J., Osbourn A., Ma P. (2019). MYB transcription factors as regulators of phenylpropanoid metabolism in plants. Mol. Plant.

[66] Lee D.H., Kim Y.K., Son Y., Park G.H., Kwon H.Y., Park Y., Park E.J., Lee S.Y., Kim H.J. (2023). Multivariate Analysis among Marker Compounds, Environmental Factors, and Fruit Quality of *Schisandra chinensis* at Different Locations in South Korea. Plants.

[67] Shang J., Zhao Q., Yan P., Sun M., Sun H., Liang H., Zhang D., Qian Z., Cui L. (2023). Environmental factors influencing potential distribution of *Schisandra sphenanthera* and its accumulation of medicinal components. Front. Plant Sci..

