# Supplementary material for: Exploratory Assessment of Geographical Variation in Metabolomic Profiles and In Vitro Bioactivities of Wild Schisandra chinensis Berries from the Changbai Mountains, China
Source: Foods. 2026 Nov 3;15(21):3941. doi: 10.3390/foods15213941 (PMC13763012; doi:10.3390/foods15213941)
Supplement: Supplementary file 1 [file foods-15-03941-s001.zip › foods-4622668-supplementary.pdf]

Supplementary Table S1: Molecular weight detection parameters of each component

Compound	Rt/min	Scan Mode	[M+H]–	MS/MS [M+H]–	RF V	CE V
Schizandrin A	5.19	ESI^+	417.2	316.1/402.1	193	24.6/21.4
Schizandrin B	7.37	ESI^+	401.1	300.1/331.1	186	25.4/18.6
Schizandrol A	1.16	ESI^+	433.1	384.1/415.2	136	20.1/10.2
Schizandrol B	1.39	ESI^+	399.1	368.1/369.1	138	19.8/16.6
schisantherin A	2.63	ESI^+	559.1	415.1/437.1	240	21.6/22.4
Schisanhenol	3.19	ESI^+	403.1	340.1/371.1	185	22.1/18.3
Gomisin D	1.24	ESI^+	531.2	401.1/485.2	124	10.2/14.3
Angeloyl gomisin H	1.82	ESI^+	501.1	401.1/483.1	129	10.2/10.2
Gomisin J	1.31	ESI^+	389.1	287.0/357.1	170	22.4/16.8

Supplementary Table S2 Data Information of Wild *Schisandra chinensis* Samples in the Changbaishan Mountain Region

No.	Longitude/E	Latitude/N	Origin
1	123.1041667	40.27916667	Self-collect
2	124.3791667	41.54583334	GBIF
3	124.4958333	40.4375	Self-collect
4	124.6625	41.3625	GBIF
5	124.7375	40.9125	GBIF
6	124.7458333	41.87916667	Self-collect
7	124.7541667	41.34583334	CVH
8	124.7625	40.95416667	NSII
9	124.7791667	40.9375	GBIF
10	124.7875	40.92083334	GBIF
11	124.7958333	40.9375	CVH
12	124.9291667	41.84583334	GBIF
13	125.2208333	41.9625	GBIF
14	125.3291667	41.32083334	Self-collect
15	125.8458333	41.74583334	Self-collect
16	125.8958333	41.52916667	NSII
17	125.9375	41.6375	NSII
18	126.5625	41.97083334	Self-collect
19	128.4791667	43.12083334	Self-collect
20	129.6291667	45.6875	Self-collect

Supplementary Table S3. Regression data for the analysis of 9 types of lignans using high-performance liquid chromatography

No.	Compound	Regression equation	R^2	Linear range (ng/mL)	LOD (ng/mL)	LQD (ng/mL)
1	Schizandrin A	Y=8102.9X-57277	0.9999	15.876-1960	4.768	14.303
2	Schizandrin B	Y=3455.4X+17084	0.9999	26.568-3280	7.978	23.935
3	Schizandrol A	Y=1976.5X+ 379556	0.9987	80.028-9880	24.032	72.097
4	Schizandrol B	Y=3092.1X+10255	0.9999	14.197-1752	4.263	12.790
5	schisantherin A	Y=265.37X+4035.1	0.9987	7.031-868	2.111	6.334
6	Schisanhenol	Y=2468.9X+5433.3	0.9997	3.013-372	0.905	2.714
7	Gomisin D	Y=899.44X-315.71	0.999	5.994-740	1.800	5.400
8	Angeloyl gomisin H	Y=1316.1X+4541.3	0.9999	13.608-1680	4.086	12.259
9	Gomisin J	Y=277.8X+1391.4	0.9998	6.383-788	1.917	5.750

R: Correlation coefficient; LOD: Limit of detection; LOQ: Limit of quantitation. Calibration curves were constructed at six concentration levels for each lignan, covering the range of X–Y ng/mL (individual ranges are given in the table).

Supplementary Table S4. Method validation for the 9 lignans of LC-MS

Peak No.	Intra-day precision [RSD%]		Inter-day precision [RSD%]		Stability [RSD%]		Repeatability [RSD%]	
	t_R	Pa	t_R	Pa	t_R	Pa	t_R	Pa
1	0.23	3.12	0.33	3.16	0.31	3.46	0.53	4.12
2	0.34	3.65	0.36	3.35	0.33	3.74	0.55	4.65
3	0.45	2.78	0.56	2.28	0.61	2.88	0.63	3.78
4	0.24	1.89	0.26	1.85	0.32	2.06	0.44	2.89
5	0.54	0.78	0.50	1.08	0.51	1.23	0.62	1.78
6	0.55	1.03	0.51	1.11	0.61	1.22	0.73	1.56
7	0.33	0.76	0.36	1.02	0.52	1.12	0.58	1.76
8	0.41	1.23	0.32	1.33	0.44	1.67	0.56	2.23
9	0.38	1.12	0.34	1.32	0.36	1.46	0.56	2.12

RSD: Relative standard deviation; tR: Retention times; Pa: Peak areas.

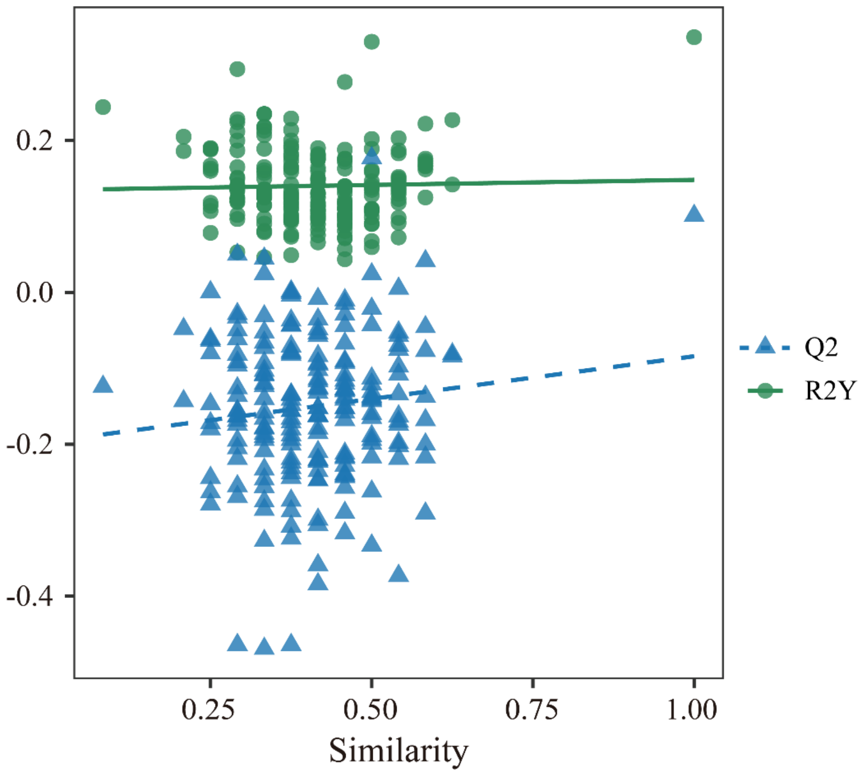

Figure S1. Permutation test (200 random permutations) for the OPLS-DA model.

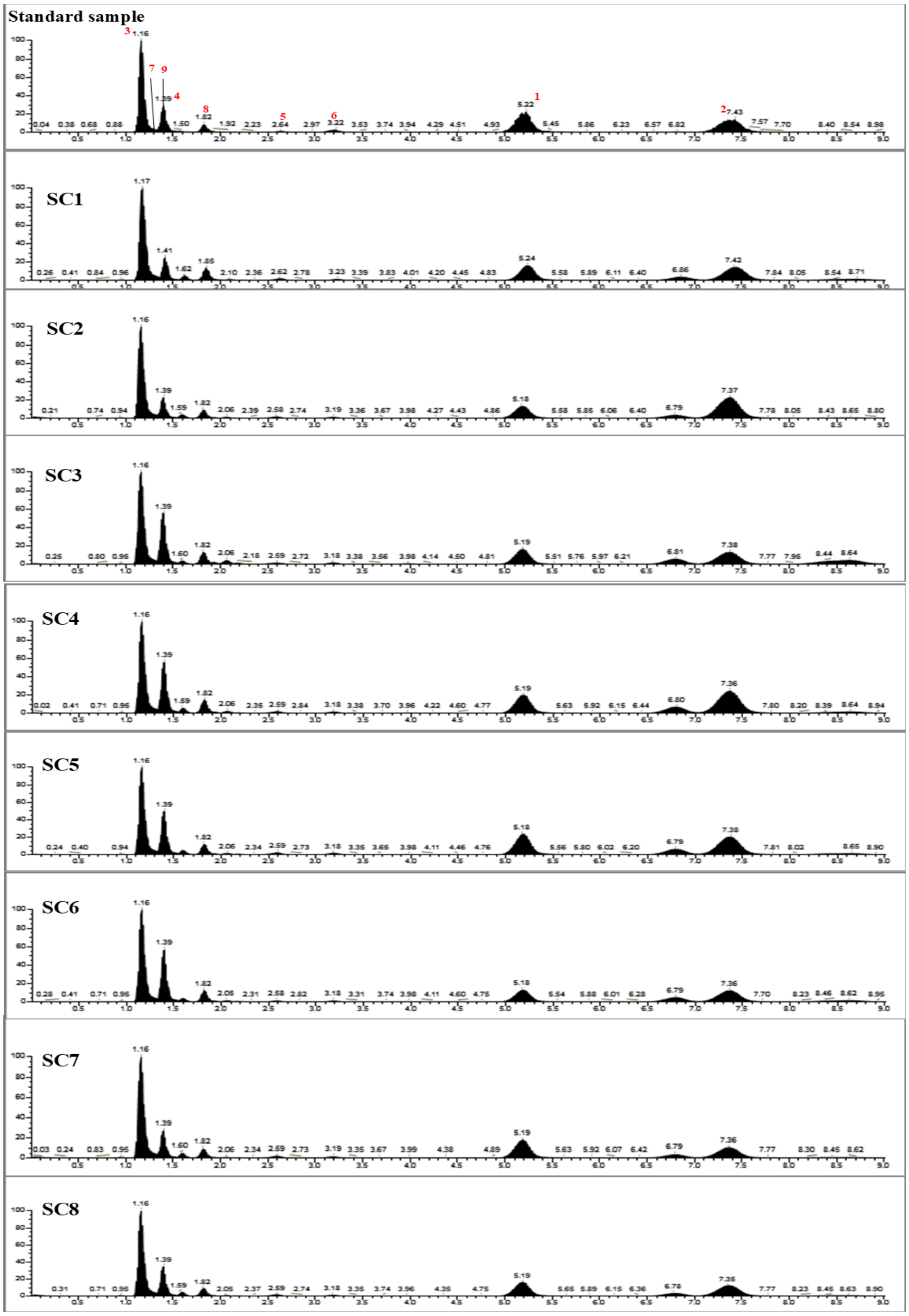

Figure S2. Standard and sample chromatograms.1:Schizandrin A,2:Schizandrin B,3:Schizandrol A,4:Schizandrol B,5:schisantherin A,6:Schisanhenol,7:Gomisin D,8:Angeloyl gomisin H,9:Gomisin J

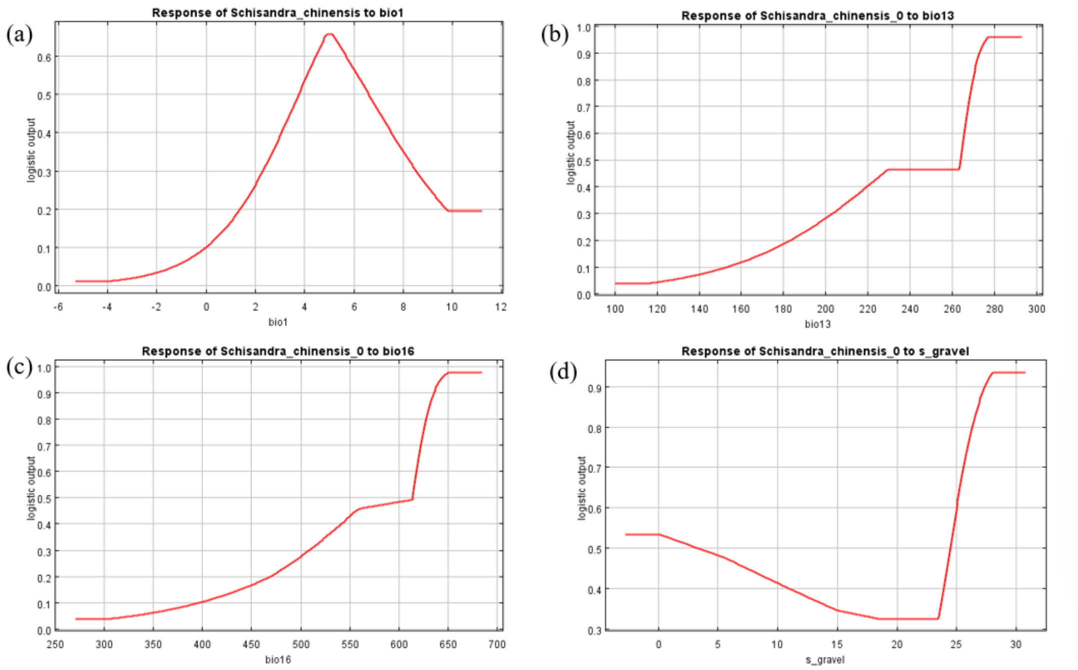

Figure S3. Response curves of the four most influential environmental variables for Schisandra chinensis based on MaxEnt single-variable models (_only). The y-axis represents the logistic output (habitat suitability), and the x-axis shows the range of each environmental variable. (a) Annual mean temperature (bio1); (b) Precipitation of the wettest month (bio13); (c) Precipitation of the wettest quarter (bio16); (d) Gravel content of the upper soil layer (t_gravel). The curves illustrate the nature of each relationship: monotonic negative (bio1), positive sigmoidal (bio13), threshold-dependent (bio16), and unimodal (t_gravel).
